# High- and low-affinity *cre* boxes for CcpA binding in *Bacillus subtilis* revealed by genome-wide analysis

**DOI:** 10.1186/1471-2164-13-401

**Published:** 2012-08-17

**Authors:** Bogumiła C Marciniak, Monika Pabijaniak, Anne de Jong, Robert Dűhring, Gerald Seidel, Wolfgang Hillen, Oscar P Kuipers

**Affiliations:** 1 Department of Molecular Genetics, Groningen Biomolecular Sciences and Biotechnology Institute, Centrum voor Levenswetenschappen, University of Groningen, Nijenborgh 7, 9747 AG, Groningen, The Netherlands; 2 Lehrstuhl für Microbiologie, Institut für Biologie der Friedrich-Alexander-Universität Erlangen-Nürnberg, Staudtstr. 5, 91058, Erlangen, Germany; 3 Kluyver Center for Genomics of Industrial Fermentation, Delft/Groningen, The Netherlands

**Keywords:** CcpA, Catabolite responsive elements (*cre*) affinity, *Cre* box motif

## Abstract

**Background:**

In *Bacillus subtilis* and its relatives carbon catabolite control, a mechanism enabling to reach maximal efficiency of carbon and energy sources metabolism, is achieved by the global regulator CcpA (carbon catabolite protein A). CcpA in a complex with HPr-Ser-P (seryl-phosphorylated form of histidine-containing protein, HPr) binds to operator sites called catabolite responsive elements, *cre*. Depending on the *cre* box position relative to the promoter, the CcpA/HPr-Ser-P complex can either act as a positive or a negative regulator. The *cre* boxes are highly degenerate semi-palindromes with a lowly conserved consensus sequence. So far, studies aimed at revealing how CcpA can bind such diverse sites were focused on the analysis of single *cre* boxes. In this study, a genome-wide analysis of *cre* sites was performed in order to identify differences in *cre* sequence and position, which determine their binding affinity.

**Results:**

The transcriptomes of *B. subtilis* cultures with three different CcpA expression levels were compared. The higher the amount of CcpA in the cells, the more operons possessing *cre* sites were differentially regulated. The *cre* boxes that mediated regulation at low CcpA levels were designated as strong (high affinity) and those which responded only to high amounts of CcpA, as weak (low affinity). Differences in the sequence and position in relation to the transcription start site between strong and weak *cre* boxes were revealed.

**Conclusions:**

Certain residues at specific positions in the *cre* box as well as, to a certain extent, a more palindromic nature of *cre* sequences and the location of *cre* in close vicinity to the transcription start site contribute to the strength of CcpA-dependent regulation. The main factors contributing to *cre* regulatory efficiencies, enabling subtle differential control of various subregulons of the CcpA regulon, are identified.

## Background

A well-known phenomenon among bacteria is the sole utilization of the most favored carbon source (e.g., glucose, fructose or malate) over other sugars present in the environment. The regulatory mechanism coordinating the metabolism of carbon and energy sources in order to maximize the metabolic efficiency is called carbon catabolite control, i.e., carbon catabolite repression (CCR) and carbon catabolite activation (CCA). Carbon catabolite control in *Bacillus subtilis* and other low-GC Gram-positive bacteria is exerted by the CcpA protein (catabolite control protein A) [1]. CcpA is a member of the LacI/GalR family of transcriptional regulators [2] and it can act either as a positive or negative regulator of genes that are in most cases involved in carbon acquisition or metabolism [3]. CcpA is synthesized constitutively, regardless to the availability of preferred carbon sources [4], it forms a dimer [5] and its activity is modulated by a complex interaction with either one of the corepressors, HPr or Crh [4-8]. In the presence of glucose or other rapidly metabolized carbon sources, the histidine-containing protein (HPr), and an HPr-like protein (Crh), are phosphorylated on a conserved Ser-46 residue by HPr kinase [9,10]. Binding of the seryl-phosphorylated HPr (HPr-Ser-P) or Crh (Crh-Ser-P) to CcpA stimulates the activity of CcpA [6-8,10]. During growth on carbohydrates there is much more HPr than Crh in the cell [11]. Notably, the Crh-specific function in the regulation of expression during growth on substrates other than carbohydrates was recently revealed [1]. Hence, Crh seems to play a secondary role in CCR. Next to HPr and Crh, low-molecular-weight molecules like NADP, glucose-6-phosphate (G6P), and fructose-1,6-bisphosphate (FBP) modulate CcpA activity by either stimulation of HPr kinase activity (FBP) [12,13], enhancement of CcpA affinity for HPr-Ser-P (FBP) [14], triggering cooperative CcpA binding to DNA (G6P) [15], or enhancing the CcpA interaction with the transcription machinery (NADP/NADPH) [16].

CcpA binds to DNA at *cis*-acting sequences called catabolite responsive elements (*cre*) located in the promoter region or within open reading frames of the regulated genes and operons. So far more than 50 *cre* sites were identified in the *B. subtilis* genome [1]. A general rule was deduced, stating that genes with *cre* boxes located upstream of −35 sequences of the promoter are subject to activation by the CcpA complex, as shown for *ackA*[17], *pta*[18]*ilvB*[19,20]. However, *ackA* is cooperatively activated by CcpA and CodY [21,22] and full activation of *ackA* requires also an additional conserved sequence present upstream of the *cre* box [23]. Moreover, the *lev* operon is subject to CcpA repression, although the *lev cre* site is located upstream of the promoter. However, regulation of the *lev* operon involves also the LevR transcriptional activator: binding of CcpA to the *lev cre* site prevents a productive interaction between LevR and RNA polymerase [24]. Binding of CcpA to *cre* boxes overlapping the promoter leads to transcriptional repression by interfering with the transcription machinery binding, as for *amyEbglPcccAdctPglpFphoPacuA*[25-31]. The binding of the protein complex to *cre* boxes that are located downstream of the transcription start site blocks transcription elongation, as is the case for most of the genes and operons regulated by CcpA [1,7].

*Cre* boxes are highly degenerate pseudo-palindromes with the consensus sequence WTGNNARCGNWWWCAW, where the strongly conserved residues are underlined [32-34]. Little is known about how CcpA can bind to such diverse *cre* sequences. Our hypothesis was that CcpA can bind with different affinities to *cre* boxes with particular sequence and/or position in relation to the transcription start site (TSS). In order to identify *cre* boxes with different affinities, CcpA expression was induced to three different levels using a tetracycline-dependent gene regulation system [35] and genome wide analysis of *cre* boxes was performed using transcriptome analyses combined with bioinformatics tools. High- and low-affinity *cre* boxes with subtle differences in their sequence and/or position in relation to the TSS are revealed.

## Results

### Tight regulation of CcpA production level

**Figure 1:**
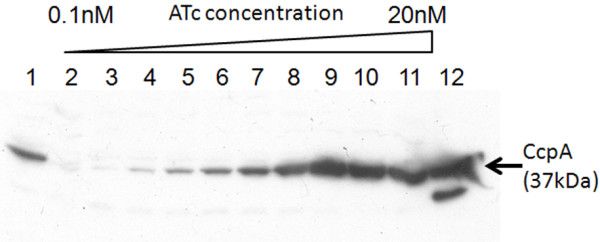
**Tight regulation of the CcpA expression level in *B. subtilis* strain MP902 (P*tet*-*ccpA*, P*xyl*-*tetR*).** Lane 1, wild type strain 168; lanes 2 – 11, MP902 grown in presence of 0.2 % xylose and increasing concentration of anhydrotetracycline (ATc): 0.1, 0.2, 04, 0.7, 1, 2, 4, 8, 10 and 20 nM, respectively; lane 12, 200 ng of purified CcpA. The representative graph of three reproducible experiments is shown.

In order to enable very tight control of the CcpA expression level in *B. subtilis*, strain MP902 (P*tet-ccpA*, P*xyl-tetR*) was constructed. Strain MP902 carries the *ccpA* gene under control of the tetracycline-inducible promoter, P*tet*, integrated in the native promoter locus and the P*tet* repressor, *tetR*, under control of the xylose-inducible promoter, P*xyl*, located on the plasmid pWH119 [35]. To show tight regulation of the CcpA expression level, the MP902 strain was grown in rich TY medium [36] supplemented with 1 % glucose, 0.2 % xylose and a wide range of concentrations (0.1 – 20 nM) of P*tet* inducer, anhydrotetracycline (ATc) which is a non-bacteriostatic tetracycline analog. As demonstrated in Figure 1, the system allows obtaining several distinct expression levels of CcpA.

**Figure 2:**
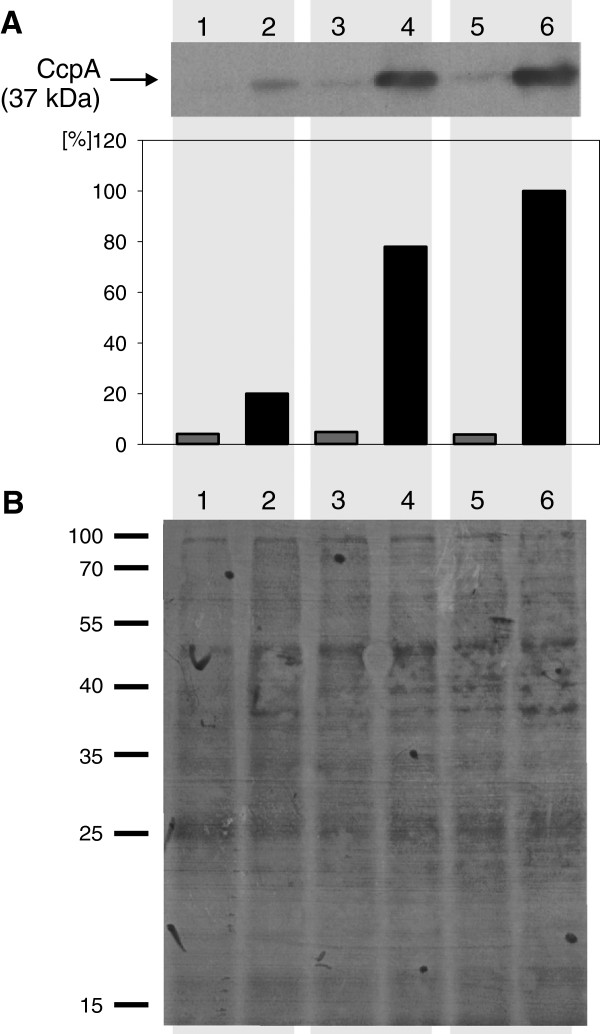
**CcpA expression levels in *B. subtilis* cultures used for DNA microarray experiments.** (**A**) *upper panel*, CcpA detection using anti-CcpA antibody; *lower panel*, signal quantification with ImageJ. Four CcpA expression levels were achieved by growing *B. subtilis* strain MP902 (P*tet*-*ccpA*, P*xyl*-*tetR*) in absence (lanes 1, 3 and 5) and in presence of 0.1, 2 and 20 nM ATc (lanes 2, 4 and 6), respectively. All cultures were grown in presence of 0.2 % xylose and 1 % glucose. Shadows in the background of the picture indicate culture pairs used in microarray experiments (**B**) Ponceau S control membrane staining for protein load verification. Lane numbers correspond to lane numbers in panel A. The representative graphs of three reproducible experiments are shown.

In order to test the influence of the different CcpA amounts in the cells on the CcpA regulon, three representative CcpA expression levels (hereafter referred to as low, medium and high) were chosen and the cultures were used for microarray experiments. For transcriptome analyses, the MP902 strain was grown in rich TY medium [36], since most likely it contains inducers for secondary regulators which could hide CCR in minimal medium and the samples were taken during exponential growth because CCR is expected to be strongest during maximal cell growth. The strain was grown in the presence of 0.2 % xylose to induce TetR expression and a high concentration of glucose (1 %) in order to ensure sufficient production of CcpA cofactors like HPr-Ser-P, NADP, glucose-6-phosphate (G6P) or fructose-1,6-bisphosphate (FBP) and optimal activity of CcpA. The medium was supplemented with different concentrations of ATc, exerting different CcpA production levels in the different cultures: 0.1 nM ATc (low CcpA induction level), 2 nM ATc (medium CcpA induction level) and 20 nM ATc (high CcpA induction level). The control culture was grown without ATc leading to no or only residual CcpA production. The CcpA production levels of the different MP902 cultures used for microarray experiments were assessed by Western blotting (Figure 2).

### Effect of different CcpA amounts on gene regulation

The transcriptional profiles of exponentially growing cells of *B. subtilis* MP902 (P*tet-ccpA*, P*xyl-tetR*) grown in rich medium supplemented with glucose and xylose and expressing CcpA at low, medium and high levels (Figure 2) due to the presence of different concentrations of the P*tet* inducer, ATc, were compared to the transcriptional profile of MP902 cells grown in the corresponding medium but without ATc (no CcpA expression induction). Our first observation was that the more CcpA present in the cells the more genes were found to be significantly regulated (Table 1). Genes were considered to be regulated if they were at least 1.8 fold up- or downregulated. When CcpA was expressed at low, medium and high levels, 128, 343 and 408 genes were found to be differentially expressed, respectively. CcpA is known to act, depending on the *cre* box position in relation to the transcriptional start site (TSS), as a repressor or activator [37-39], but many more cases of repression than of activation are known [40]. Consistently, most of the regulated genes found in the microarray analyses with different CcpA induction levels were downregulated. For the list of expression fold changes of all the genes in the *B. subtilis* genome in all the three microarray experiments see Additional file 1 in the Supplementary Material.

**Table 1:** **Number of analyzed predicted* cre* boxes and regulated genes in response to different CcpA expression levels**

	**Level of the CcpA expression**		
	**Low (0.1 nM ATc)**	**Medium (2 nM ATc)**	**High (20 nM ATc)**
N^o^ of all genes	4106	4106	4106
N^o^ of regulated genes	128	343	408
N^o^ all predicted *cre* boxes	418	418	418
N^o^*cre* boxes of operons ^*a*^	161	161	161
N^o^ regulated operons with *cre* box ^*a*^	30	58	67

^*a*^*cre* boxes within −500 and +100 nucleotides from start codon of first genes of operons.

The first genes of operons known from the literature to possess *cre* boxes (DataBase of Transcriptional Regulation in *Bacillus subtilis*, DBTBS [41] and reviewed by Fujita [1]) and which were differentially expressed at least under the high CcpA production level were extracted from the microarray data. Since it is estimated that the CcpA regulon includes more members than known so far [1], as also shown recently [42], a prediction of putative *cre* boxes was performed. Using Genome2D [43] and a list of described *cre* boxes in the literature (reviewed by Fujita [1]) a Weight Matrix of *cre* boxes was generated: T_1_G_2_A_3_A_4_A_5_R_6_C_7_G_8_Y_9_T_10_W_11_W_12_C_13_A_14_. This *cre* motif was used to search the whole *B. subtilis* genome for putative *cre* boxes. As a result, 418 putative *cre* boxes were found: 200 in the upper and 218 in the lower strand (Table 1 and, for the complete list of found *cre*, Additional file 2). Most of the predicted *cre* boxes may not be functional taking into account their large distance from the promoter. Therefore, *cre* boxes located within −500 and +100 nucleotides relative to the start codon of the first gene of an operon were extracted. There were 161 genes possessing *cre* boxes that met these criteria (Table 1 and, for the complete list, Additional file 3). Since the search did not entirely cover the list of the known *cre* sites (for review see [1]), *cre* sites known from literature were also added to the analyzed *cre* sites. In total, there were 30, 58 and 67 operons possessing (known and predicted) *cre* sites and which were significantly downregulated under low, medium or high CcpA induction level, respectively. Three operons with known and predicted *cre* sites were activated under all these conditions (Table 2 and, in more detail, Additional file 4). For the sequences of the regions between −500 and +100 nucleotides from the start codon of first genes of operons possessing *cre* boxes that were analyzed in this study, see Additional file 5. The increase in amount of CcpA-regulated operons upon increasing amounts of CcpA indicates the presence of high-affinity *cre* boxes titrating away CcpA from the weaker *cre* boxes, which can trigger regulation of additional genes only when more functional CcpA is present in the cell. Therefore, the 31 *cre* boxes of the 30 operons (*iol operon* possesses two *cre* boxes: within *iolA* and *iolB*) repressed when CcpA was present in low amounts were designated as strong (high affinity to CcpA) and the other 38 *cre* sites of 37 operons (*gntR* possesses two *cre* sites), which were repressed only in the presence of higher amounts of CcpA in the cells (medium and high CcpA induction levels), were designated as weak (low affinity to CcpA) (Table 2). The high- and low-affinity, and the three activating *cre* boxes (Table 2) were analyzed with respect to their sequence and their position relative to the TSS. The term ‘affinity’ in this study is contractual, as direct binding assays were not performed in this study, and it is used to denote hierarchy in CcpA target genes regulation. From other (mutational) studies it is however apparent that strong regulation commonly coincides with high affinity and vice versa, so the term affinity appears to be adequate to describe differences in strong or weak regulation.

**Table 2:** **High- and low-affinity* cre* boxes of the first genes of operons**

				**Gene expression fold change**^***a***^			
	**Gene**	**Strand**	***cre*sequence**	**Low CcpA induction**	**Medium CcpA induction**	**High CcpA induction**	***cre*box to TSS distance**^***b***^
*High affinity cre boxes*							
1	*acoR*	upper	TGAAAGCGCTTTAT	**−4.8**	**−18.7**	**−21.7**	−27
2	*acsA*	lower	TGAAAGCGTTACCA	**−2.3**	**−2.5**	**−2.7**	+44
3	*acuA*	upper	TGAAAACGCTTTAT	**−2.2**	**−4.6**	**−7.7**	−26
4	*amyE*	upper	TGTAAGCGTTAACA	**−2.6**	**−10.7**	**−12.9**	+4
5	*bglP*	lower	TGAAAGCGTTGACA	**−2.5**	**−4.7**	**−4.6**	−36
6	*cccA*	lower	TGTAAGCGTATACA	**−2.2**	**−1.8**	**−2.8**	−29
7	*citM*	upper	TGTAAGCGGATTCA	**−2.6**	**−2.7**	**−2.9**	+46
8	*cstA*	lower	TGAATGCGGTTACA	**−2.2**	**−1.9**	**−2.4**	+32
9	*dctP*	upper	TGAAAACGCTATCA	**−7.4**	**−12.3**	**−16.6**	−14
10	*glpF*	upper	TGACACCGCTTTCA	**−4.3**	**−21.9**	**−35.6**	−27
11	*gmuB*	upper	TGTAAGCGTTTTAA	**−3.0**	**−15.6**	**−35.8**	+6
12	*iolA-1*	lower	TGAAAGCGTTTAAT	**−1.8**	**−1.9**	**−2.1**	+93
13	*iolA-2 (within iolB)*	lower	TGAAAACGTTGTCA	**−2.2**	**−2.5**	**−2.4**	+2404
14	*manR*	upper	TGTAAACGGTTTCT	**−2.0**	**−3.7**	**−8.0**	0
15	*msmX*	lower	AGAAAGCGTTTACA	**−2.0**	**−2.6**	**−3.1**	−15
16	*rbsR*	upper	TGTAAACGGTTACA	**−6.7**	**−15.2**	**−23.1**	+6
17	*rocG*	lower	TTAAAGCGCTTACA	**−2.6**	**−3.5**	**−3.1**	+43
18	*sacP*	lower	CGAAAACGCTATCA	**−2.1**	**−7.9**	**−8.1**	−19
19	*sucC*	upper	TGAAAGCGCAGTCT	**−2.0**	**−5.8**	**−3.4**	0
20	*treP*	upper	TGAAAACGCTTGCA	**−3.2**	**−13.0**	**−17.5**	+372
21	*uxaC*	upper	TGAAAGCGTTATCA	**−2.5**	**−3.7**	**−8.9**	+1237
22	*xsa*	lower	TAAAAGCGCTTACA	**−1.9**	**−1.8**	**−2.6**	+7
23	*xylA*	upper	TGGAAGCGCAAACA	**−2.4**	**−11.9**	**−11.1**	+144
24	*xynP*	upper	TGAAAGCGCTTTTA	**−4.0**	**−11.0**	**−17.9**	+230
25	*yisS*	upper	AGAAAACGCTTTCT	**−1.9**	**−3.5**	**−3.7**	+74
26	*yjmD*	upper	TGAAAGCGGTTCAA	**−2.2**	**−2.4**	**−8.8**	ND
27	*ykoM*	upper	TGCAAGGGCTTTCA	**−2.0**	**−3.4**	**−3.5**	+150
28	*yrpD*	upper	TGATAGCGTTTTCT	**−1.9**	**−8.0**	**−6.8**	+127
29	*ytkA*	lower	TGTAAGCGTTTGCT	**−1.9**	**−6.4**	**−6.8**	ND
30	*yulD*	lower	TGAAAGCGCTATCT	**−2.3**	**−4.9**	**−5.3**	ND
31	*yvfK*	lower	TTAAAGCGCTTTCA	**−4.0**	**−6.1**	**−10.6**	+5
*Low affinity cre boxes*							
1	*abnA*	lower	TGTAAGCGCTTTCT	−1.8	−1.7	**−2.5**	+85
2	*acoA*	lower	TGTAAGCGTTTGCT	−1.1	−1.0	**−1.8**	+462
3	*citZ*	lower	TGTAAGCATTTTCT	−1.5	**−1.8**	**−2.1**	+88
4	*csbX*	lower	TGAAAACGGTGCCA	−1.4	**−2.8**	**−2.1**	−401
5	*cydA*	lower	TGAAATGAATCGTT	1.6	1.0	**−2.7**	−21
6	*drm*	lower	TGAAAACGGTTTAT	−1.3	**−3.6**	**−3.2**	−16
7	*gntR-1*	upper	TGAAAGTGTTTGCA	−1.3	**−2.8**	**−3.2**	−41
8	*gntR-2*	upper	TGAAAGCGGTACCA	−1.3	**−2.8**	**−3.2**	+148
9	*hutP*	upper	TGAAACCGCTTCCA	−1.3	**−1.9**	**−2.6**	+209
10	*lcfA*	lower	TGAAAACGTTATCA	−1.4	**−2.6**	**−2.6**	+450
11	*levD*	lower	TGAAAACGCTTAAC	−1.5	−1.2	**−2.2**	−45
12	*malA*	upper	TGTAAACGTTATCA	−1.7	**−2.0**	**−2.6**	+6
13	*mleN*	lower	TGAAAGCGTTTTAG	−1.5	**−3.5**	**−2.4**	+21
14	*msmR*	upper	TGTAACCGCTTACT	−1.7	**−4.2**	**−12.2**	−28
15	*mtlR*	upper	TGAAAGCGTTTTAT	−1.5	**−2.7**	**−2.5**	−16
16	*odhA*	lower	TGGAAGCGTTTTTA	−1.6	**−6.6**	**−3.4**	+21
17	*pbuG*	upper	TGAAAACGTTTTTT	−1.1	−1.5	**−1.9**	+245
18	*pta*	lower	TGAAAGCGCTATAA	1.3	**−3.2**	**−2.7**	−55
19	*resA*	lower	TAAAAACGCTTTCT	−1.1	**−1.9**	**−1.9**	−72
20	*sigL*	lower	GGAAAACGCTTTCA	−1.1	**−3.1**	**−3.3**	ND
21	*wprA*	upper	TGTAAGCGGTATCT	−1.6	**−5.5**	**−4.2**	+43
22	*yckB*	lower	TGAAAACGCGATCA	−1.4	**−3.5**	**−2.1**	−48
23	*ycsA*	upper	AGAAAGCGCTTACG	−1.7	**−6.0**	**−10.3**	+67
24	*ydzA*	lower	TGAAAACGTGTCCA	−1.3	**−6.4**	**−6.4**	+9
25	*yesL*	upper	TGAAAGCGTTTTCC	−1.3	−1.6	**−2.0**	+125
26	*yfiG*	upper	AGAAAGCGGTTACA	−1.6	**−2.7**	**−4.6**	+38
27	*yncC*	upper	TGTAAACGGTTACA	−1.3	**−2.4**	**−3.8**	+84
28	*yojA*	lower	TGAAAGCGCTTTCT	1.1	−1.5	**−1.8**	+57
29	*yqgW*	upper	TGAAAACGCTATCG	−1.1	**−4.5**	**−4.2**	−39
30	*yqgY*	upper	TGAAAATGTTTACA	−1.4	**−5.4**	**−4.1**	−38
31	*ysbA*	lower	TGTAAGCGCTTTAT	1.0	**−3.8**	**−7.6**	ND
32	*ysfC*	upper	TGAAAGCGTTTTTT	−1.5	−1.5	**−2.0**	+196
33	*yugN*	lower	TGAATGCGCTTTCT	−1.7	**−2.4**	**−2.3**	ND
34	*yuxG*	lower	TGAAAACGGATACA	−1.2	**−4.2**	**−6.1**	0
35	*yvdG*	lower	TGTAACCGCTTTCT	−1.4	−1.5	**−2.1**	−28
36	*yxlH*	upper	TTGAAACGCTTTCA	−1.4	**−2.0**	**−2.3**	+260
37	*yydK*	upper	TGTAAGCGGTTTAT	−1.5	**−3.2**	**−2.4**	−21
38	*yyzE*	lower	TGAAAGCGTAACCA	−1.2	**−3.0**	**−2.1**	0
*Activating cre boxes*							
1	*ilvB*	lower	TGAAAGCGTATACA	**3.0**	**6.2**	**2.7**	+88
2	*opuE*	lower	TGAAAGCGTTTTAT	**2.3**	**2.5**	**2.3**	−103
3	*ycbP*	lower	TGAAAGCGCTCGCT	**2.5**	**3.3**	**2.6**	+30

^*a*^ In bold – genes significantly regulated (1.8 < fold < −1.8).

^***b***^*cre* box distance to transcriptional start site calculated from the conserved G residue in the middle CpG of the *cre* box.

### Analysis of *cre* box affinities in relation to their sequence

**Figure 3:**
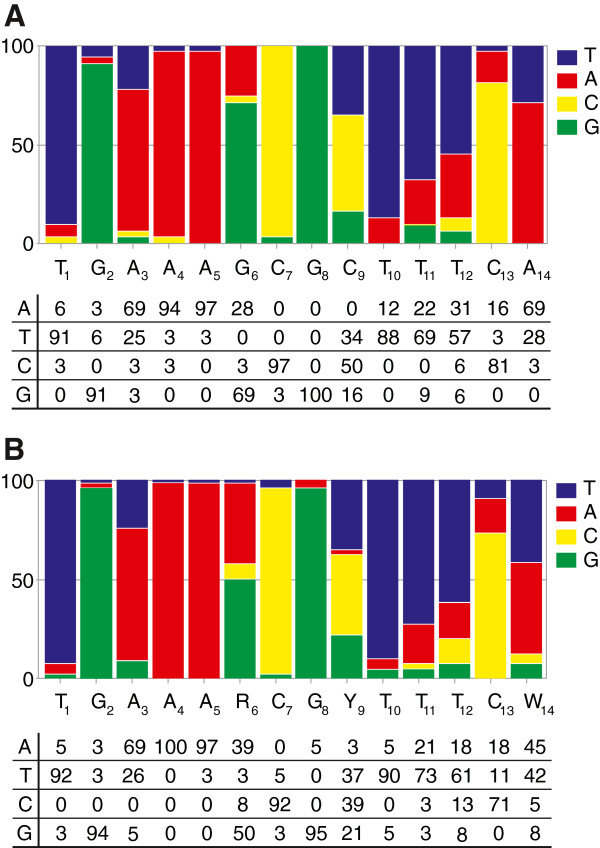
**Analysis of high- (A) and low-affinity (B) *cre* boxes responsible for gene repression.** Weight Matrix (*upper panels*) and *cre* box consensus with Position Frequency Matrix (PFM) (*lower panels*). In the consensus sequence: R is A or G, Y is T or C.

In order to detect differences within the sequence between different *cre* boxes, which putatively determine the *cre* box affinity, separate Weight Matrices for high- and low-affinity *cre* boxes that are responsible for gene repression were generated using Genome2D [43] (Figure 3). The resulting consensus sequences are T_1_G_2_A_3_A_4_A_5_G_6_C_7_G_8_C_9_T_10_T_11_T_12_C_13_A_14_ and T_1_G_2_A_3_A_4_A_5_R_6_C_7_G_8_Y_9_T_10_T_11_T_12_C_13_W_14_, for strong and weak *cre* boxes, respectively. *Cre* boxes from both groups have very conserved G_2_, C_7_ and G_8_ residues, as in *cre* motifs proposed before [32-34]. Although the differences between high- and low-affinity *cre* are not very pronounced, the *cre* boxes with high affinity to CcpA seem to have a more conserved sequence around the middle CpG (conserved GCpGC instead of RCpGY) and at the C_13_ and A_14_ positions (Figure 3). To analyze the differences in the *cre* sequences in more detail, the high- and low-affinity *cre* boxes were aligned. The alignments show that the strong *cre* boxes (Table 3) have, on average, more palindromic residues than the weak *cre* boxes (Table 4) particularly at the external residues and in the middle CpG.

**Table 3:** **Analysis of* cre* boxes with apparent high affinity to CcpA**

**Gene**	**Cre sequence**^***a***^														**Score**^***b***^
	**1**	**2**	**3**	**4**	**5**	**6**	**7**	**8**	**9**	**10**	**11**	**12**	**13**	**14**	
***acoR***	T	G	**A**	**A**	**A**	**G**	**C**	**G**	**C**	**T**	**T**	**T**	A	T	5
***acsA***	**T**	**G**	A	A	**A**	G	**C**	**G**	T	**T**	A	C	**C**	**A**	4
***acuA***	T	G	**A**	**A**	**A**	A	**C**	**G**	C	**T**	**T**	**T**	A	T	4
***amyE***	**T**	**G**	**T**	A	**A**	G	**C**	**G**	T	**T**	A	**A**	**C**	**A**	5
***bglP***	**T**	**G**	A	A	**A**	G	**C**	**G**	T	**T**	G	A	**C**	**A**	4
***cccA***	**T**	**G**	**T**	**A**	A	G	**C**	**G**	T	A	**T**	**A**	**C**	**A**	5
***citM***	**T**	**G**	T	**A**	A	G	**C**	**G**	G	A	**T**	T	**C**	**A**	4
***cstA***	**T**	**G**	A	**A**	T	G	**C**	**G**	G	T	**T**	A	**C**	**A**	4
***dctP***	**T**	**G**	**A**	A	**A**	A	**C**	**G**	C	**T**	A	**T**	**C**	**A**	5
***glpF***	**T**	**G**	**A**	C	**A**	C	**C**	**G**	C	**T**	T	**T**	**C**	**A**	5
***gmuB***	**T**	G	T	**A**	**A**	G	**C**	**G**	T	**T**	**T**	T	A	**A**	4
***iolA-1***	T	G	A	**A**	**A**	G	**C**	**G**	T	**T**	**T**	A	A	T	3
***iolA-2***	**T**	**G**	**A**	A	**A**	**A**	**C**	**G**	**T**	**T**	G	**T**	**C**	**A**	6
***manR***	T	**G**	T	**A**	**A**	A	**C**	**G**	G	**T**	**T**	T	**C**	T	4
***msmX***	A	**G**	A	**A**	**A**	G	**C**	**G**	T	**T**	**T**	A	**C**	A	4
***rbsR***	**T**	**G**	**T**	**A**	**A**	A	**C**	**G**	G	**T**	**T**	**A**	**C**	**A**	6
***rocG***	**T**	T	A	**A**	**A**	**G**	**C**	**G**	**C**	**T**	**T**	A	C	**A**	5
***sacP***	C	**G**	**A**	A	**A**	A	**C**	**G**	C	**T**	A	**T**	**C**	A	4
***sucC***	T	**G**	**A**	A	A	**G**	**C**	**G**	**C**	A	G	**T**	**C**	T	4
***treP***	**T**	**G**	A	**A**	**A**	A	**C**	**G**	C	**T**	**T**	G	**C**	**A**	5
***uxaC***	**T**	**G**	**A**	A	**A**	G	**C**	**G**	T	**T**	A	**T**	**C**	**A**	5
***xsa***	**T**	A	A	**A**	**A**	**G**	**C**	**G**	**C**	**T**	**T**	A	C	**A**	5
***xylA***	**T**	**G**	G	A	A	**G**	**C**	**G**	**C**	A	A	A	**C**	**A**	4
***xynP***	**T**	G	**A**	**A**	**A**	**G**	**C**	**G**	**C**	**T**	**T**	**T**	T	**A**	6
***yisS***	**A**	**G**	**A**	**A**	**A**	A	**C**	**G**	C	**T**	**T**	**T**	**C**	**T**	6
***yjmD***	**T**	G	A	**A**	**A**	G	**C**	**G**	G	**T**	**T**	C	A	**A**	4
***ykoM***	**T**	**G**	C	**A**	**A**	**G**	G	G	**C**	**T**	**T**	T	**C**	**A**	5
***yrpD***	T	**G**	**A**	T	**A**	G	**C**	**G**	T	**T**	T	**T**	**C**	T	4
***ytkA***	T	**G**	T	**A**	**A**	G	**C**	**G**	T	**T**	**T**	G	**C**	T	4
***yulD***	T	**G**	**A**	A	**A**	**G**	**C**	**G**	**C**	**T**	A	**T**	**C**	T	5
***yvfK***	**T**	T	**A**	**A**	**A**	**G**	**C**	**G**	**C**	**T**	**T**	**T**	C	**A**	6
**palindrome %**^*c*^	68	71	52	61	84	32	97	97	32	84	61	52	71	68	Average score = 4.6

^*a*^ In bold – palindromic residues.

^*b*^ Score - a number of palindromic pairs.

^*c*^ Occurrence of palindromic residue at each position.

*Cre* boxes of repressed genes are aligned.

**Table 4:** **Analysis of* cre* boxes with apparent low affinity to CcpA**

**Gene**	**Cre sequence**^***a***^														**Score**^***b***^
	**1**	**2**	**3**	**4**	**5**	**6**	**7**	**8**	**9**	**10**	**11**	**12**	**13**	**14**	
***abnA***	T	**G**	T	**A**	**A**	**G**	**C**	**G**	**C**	**T**	**T**	T	**C**	T	5
***acoA***	T	**G**	T	**A**	**A**	G	**C**	**G**	T	**T**	**T**	G	**C**	T	4
***citZ***	T	**G**	T	**A**	**A**	G	C	A	T	**T**	**T**	T	**C**	T	3
***csbX***	**T**	**G**	A	A	**A**	A	**C**	**G**	G	**T**	G	C	**C**	**A**	4
***cydA***	T	G	A	A	**A**	**T**	G	A	**A**	**T**	C	G	T	T	2
***drm***	T	G	**A**	**A**	**A**	A	**C**	**G**	G	**T**	**T**	**T**	A	T	4
***gntR-1***	**T**	**G**	A	**A**	**A**	G	T	G	T	**T**	**T**	G	**C**	**A**	4
***gntR-2***	**T**	**G**	A	A	**A**	G	**C**	**G**	G	**T**	A	C	**C**	**A**	4
***hutP***	**T**	**G**	A	**A**	**A**	C	**C**	**G**	C	**T**	**T**	C	**C**	**A**	5
***lcfA***	**T**	**G**	**A**	A	**A**	**A**	**C**	**G**	**T**	**T**	A	**T**	**C**	**A**	6
***levD***	T	G	A	**A**	**A**	A	**C**	**G**	C	**T**	**T**	A	A	C	3
***malA***	**T**	**G**	T	A	**A**	**A**	**C**	**G**	**T**	**T**	A	T	**C**	**A**	5
***mleN***	T	G	**A**	**A**	**A**	G	**C**	**G**	T	**T**	**T**	**T**	A	G	4
***msmR***	T	**G**	**T**	**A**	**A**	C	**C**	**G**	C	**T**	**T**	**A**	**C**	T	5
***mtlR***	T	G	**A**	**A**	**A**	G	**C**	**G**	T	**T**	**T**	**T**	A	T	4
***odhA***	**T**	G	G	**A**	**A**	G	**C**	**G**	T	**T**	**T**	T	T	**A**	4
***pbuG***	T	G	**A**	**A**	**A**	**A**	**C**	**G**	**T**	**T**	**T**	**T**	T	T	5
***pta***	**T**	G	**A**	A	**A**	**G**	**C**	**G**	**C**	**T**	A	**T**	A	**A**	5
***resA***	T	A	**A**	**A**	**A**	A	**C**	**G**	C	**T**	**T**	**T**	C	T	4
***sigL***	G	**G**	**A**	**A**	**A**	A	**C**	**G**	C	**T**	**T**	**T**	**C**	A	5
***wprA***	T	**G**	T	A	**A**	G	**C**	**G**	G	**T**	A	T	**C**	T	3
***yckB***	**T**	**G**	**A**	A	A	A	**C**	**G**	C	G	A	**T**	**C**	**A**	4
***ycsA***	A	**G**	A	**A**	**A**	**G**	**C**	**G**	**C**	**T**	**T**	A	**C**	G	5
***ydzA***	**T**	**G**	A	**A**	A	**A**	**C**	**G**	**T**	G	**T**	C	**C**	**A**	5
***yesL***	T	**G**	**A**	**A**	**A**	G	**C**	**G**	T	**T**	**T**	**T**	**C**	C	4
***yfiG***	A	**G**	A	**A**	**A**	G	**C**	**G**	G	**T**	**T**	A	**C**	A	4
***yncC***	**T**	**G**	**T**	**A**	**A**	A	**C**	**G**	G	**T**	**T**	**A**	**C**	**A**	6
***yojA***	T	**G**	**A**	**A**	**A**	**G**	**C**	**G**	**C**	**T**	**T**	**T**	**C**	T	6
***yqgW***	T	**G**	**A**	A	**A**	A	**C**	**G**	C	**T**	A	**T**	**C**	G	4
***yqgY***	**T**	**G**	A	**A**	**A**	**A**	T	G	**T**	**T**	**T**	A	**C**	**A**	5
***ysbA***	T	G	T	**A**	**A**	**G**	**C**	**G**	**C**	**T**	**T**	T	A	T	4
***ysfC***	T	G	**A**	**A**	**A**	G	**C**	**G**	T	**T**	**T**	**T**	T	T	4
***yugN***	T	**G**	**A**	**A**	T	**G**	**C**	**G**	**C**	T	**T**	**T**	**C**	T	5
***yuxG***	**T**	**G**	A	**A**	A	A	**C**	**G**	G	A	**T**	A	**C**	**A**	4
***yvdG***	T	**G**	T	**A**	**A**	C	**C**	**G**	C	**T**	**T**	T	**C**	T	5
***yxlH***	**T**	T	G	**A**	**A**	A	**C**	**G**	C	**T**	**T**	T	C	**A**	4
***yydK***	T	G	T	**A**	**A**	G	**C**	**G**	G	**T**	**T**	T	A	T	3
***yyzE***	**T**	**G**	A	A	A	G	**C**	**G**	T	A	A	C	**C**	**A**	3
**palindrome %**^*c*^	40	66	42	74	87	32	90	90	32	87	74	42	66	40	Average score = 4.3

^*a*^ In bold – palindromic residues.

^*b*^ Score - a number of palindromic pairs.

^*c*^ Occurrence of palindromic residue at each position.

*Cre* boxes of repressed genes are aligned.

The *cre* sites of the genes that were activated in this study (*ilvBopuE* and *ycbP*) were not included in the Weight Matrix generation nor *cre* alignment as *cre* boxes that are responsible for gene expression activation may need additional (upstream) sequences, as shown for instance for *ackA*[23]. Moreover, their sequence might putatively differ from the repressing *cre* sites, but the population of activating *cre* sites is too small to perform statistically significant analysis. However, taking into account the fact that all three genes that were activated in the microarray experiments in this study are regulated already in presence of low amounts of CcpA in the cell, the activating *cre* sites seem to take a higher place in the hierarchy of the genes regulated by CcpA. Additionally, the *cre* sites of *ilvB* and *ycbP* appear to match the consensus of the high-affinity *cre* boxes better compared to the consensus of low-affinity *cre* boxes (Table 2).

### Analysis of the influence of relative *cre* box position on regulation

**Figure 4:**
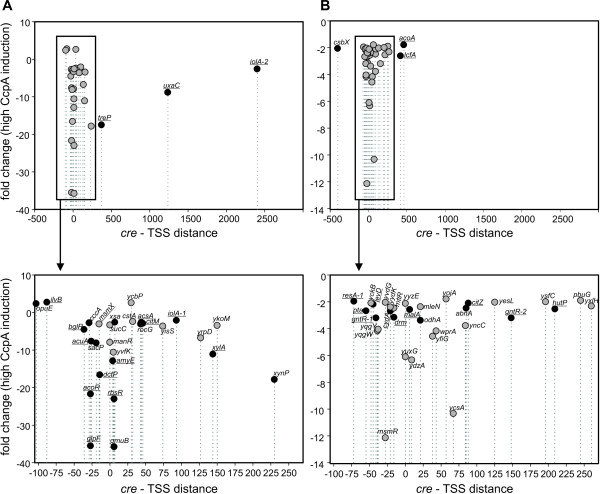
**Correlation between the *cre* to TSS distance to corresponding gene expression level (fold change).** (**A**) High-affinity *cre* boxes. (**B**) Low-affinity *cre* boxes. *Black circles* - *cre* boxes of the genes for which TSSs were detected experimentally; *grey circles* - *cre* boxes of the genes for which TSSs were predicted in this study, underlined gene names – genes with *cre* sites known from literature. “0” on the X ax represents the TSS position, negative numbers – *cre* boxes upstream TSS, positive numbers – *cre* boxes downstream TSS. For clarity, the outliers were removed (for the full list of *cre*-TSS distance, see Additional file 5).

To find out whether the *cre* box position in relation to the promoter plays a role in determining the affinity to CcpA, the distance between *cre* boxes and the corresponding transcription start sites (TSS) was analyzed. The TSS of the regulated genes possessing a *cre* box were extracted from the literature or, when this information was lacking, predicted in this study (Table 2 and Additional file 5). The calculated *cre* to TSS distance (counting from the conserved G residue in the middle of the *cre* boxes to the TSS) was plotted against expression level fold change of the regulated genes under high levels of CcpA, separately for the genes with either high (Figure 4A) and low affinity *cre* boxes (Figure 4B). The majority of high affinity *cre* boxes are localized in close vicinity to the TSS (*cre*-TSS distance from 0 to +7, that is a TSS within the *cre* box) and around positions −27, -14 and +44. Repression of the genes with *cre* sites located with increment of approximately 10–11 nt (full helix turn) was significantly stronger, such as found for *cre* boxes of *acoR*, *glpF*, *dctP*, *gmuB*, *xynP*, *treP*, which are localized at positions −27, -27, -14, +6, +230, +372, respectively. Further downstream from the TSS, there are more low affinity *cre* boxes than high affinity ones.

## Discussion

CcpA is a global regulator of carbon catabolism [3] controlling expression of genes by binding to cognate operator sequences, *cre*, which is characterized by a low-conserved consensus sequence [32-34]. Hence, it seems possible that CcpA binds some *cre* sites with higher affinity than others. So far, the global studies of CcpA-dependent carbon catabolite repression were focused on identification of the members of the CcpA regulon [40,42,44], while the analysis of *cre* boxes in respect to their sequences, position and affinities in CcpA binding have been focused only on single examples [7,17,33,34,45]. A broader comparison of 32 *cre* boxes sequences and function was published by Miwa Y. *et al*. and it was deduced that a lower mismatching of *cre* sequences to the query sequence in the same direction as that of transcription of the target genes and a more palindromic sequence of *cre* boxes are desirable for their better function [33]. The goal of our study was to perform a genome-wide analysis of *cre* boxes in order to reveal *cre* boxes with high and low binding affinities by comparing the CcpA regulon under three distinct conditions, where different amounts of CcpA were present in the cells and to identify *cre* features that determine this affinity.

Using a tetracycline-dependent gene regulation system [35] we achieved a tightly-controlled *ccpA* expression, leading to a wide range of CcpA amounts in the cells. *B. subtilis* cultures with relative low, medium or high amounts of CcpA in the cells were subjected to transcriptome analyses. The cells were grown in the presence of glucose to ensure sufficient production of low-molecular-weight modulators of CcpA activity (NADP, glucose-6-phosphate, fructose-1,6-bisphosphate). As expected, higher levels of CcpA protein lead to more genes significantly up- or downregulated. Most of the regulated genes, however, were affected indirectly, as they were lacking a *cre* site. Genes regulated indirectly in a CcpA-dependent manner (no *cre* or unfunctional *cre*) were already observed before and were proposed to be grouped in class II, next to class I that includes genes regulated by CcpA directly [40,46,47]. In our analysis, only genes belonging to class I were taken into account as the subject of this study was the nature of discriminating *cre* boxes. Many repressed genes are σA-dependent and do not need another inducing protein for their expression. However, expression of some genes is regulated by more than one regulator. In those rare cases of multiple regulation, the full extent of regulation would not be observed in our transcriptome analysis, but this does not affect our analysis since we are looking at the relative strength of repression at different CcpA concentrations.

The search for putative *cre* boxes in the *B. subtilis* genome, using a *cre* motif generated from the *cre* boxes known from DBTBS [41], T_1_G_2_A_3_A_4_A_5_R_6_C_7_G_8_Y_9_T_10_W_11_W_12_C_13_A_14_, resulted in 418 putative *cre* boxes. The majority of the predicted *cre* boxes were within ORFs far away from promoters and, although functional *cre* boxes located within coding sequences are present in the *B. subtilis* genome, a lot of the predicted *cre* sites seemed to be at a too large distance from the promoter to possibly be able to play a role in regulation of gene expression. Therefore, *cre* boxes located within −500 and +100 nucleotides from the first nucleotide of a start codon of the first genes of an operon were extracted. Also *cre* boxes triggering gene regulation that are known from the literature, but not predicted by our method, were included in our analysis. The genes differentially expressed at least at a high CcpA production level and possessing *cre* box(es) known from literature [1,41] and/or predicted in this study were selected. Among the selected genes, 30 were downregulated and 3 were upregulated at a low CcpA induction level, while the other 37 genes were downregulated only when CcpA was produced at higher levels (medium and high CcpA induction levels). For all these genes, expression fold changes were calculated as ratios of the amounts of transcripts downstream of *cre* boxes as the microarray chip probes were synthesized upstream from them. Of the regulated first genes of operons possessing known and/or predicted *cre* box, chip probes of only *kdgR* and *resA* were upstream from *kdgRcre* and second *cre* of *resA* (located 1709 bp downstream from TSS). Therefore, these *cre* boxes were not included in the sequence and position analysis of *cre* boxes. Since regulation depends on CcpA-*cre* binding, *cre* boxes causing significant regulation of downstream operons already when a small amount of CcpA is available are supposed to have a high affinity to CcpA and titrate CcpA away from low-affinity *cre* sites, which are able to exert regulation of other operons only when more CcpA is present. Notably, over a dozen of known *cre’s* fell out of our data set, because the corresponding genes were not significantly regulated in any of the three microarray experiments. Despite of the fact that they could be considered as very low-affinity sites, they were not included in the analysis as lack of the differential expression might have been a false negative result due to, e.g., high background signal, bad spot quality on the microarray slides, mRNA degradation, growth conditions, more complex regulation or yet unidentified factors. Moreover, it should be noted that division of *cre* boxes to two affinity groups is a simplification necessary for this analysis. Very likely a gradient distribution of *cre* site affinities occurs in nature, which would be difficult to assess.

The detailed analysis of the sequences of high- and low-affinity *cre* boxes, led to a few interesting observations. The G_2_ and middle C_7_ and G_8_ residues (Figure 3), known as highly conserved residues [32-34] are conserved in both high- and low-affinity *cre* boxes. Interestingly, the high-affinity *cre* boxes have more conserved G_6_ and C_9_ surrounding the middle CpG and C_13_ (palindromic to the conserved G_2_) and A_14_ (palindromic to T_1_) and their sequences are significantly more palindromic overall. It was observed before that a more palindromic sequence of *cre* sites contributes to a better function [33]. The more palindromic nature of the high-affinity *cre* sites (in comparison low-affinity *cre* sites) might create a more symmetric DNA conformation, preferred for CcpA binding. Although the bases at positions 4 and 11 are more often palindromic to each other in the weak *cre* boxes, this is obviously less important for the *cre* strength. In a previous study [34] it was shown that CcpA binds with similar affinities to different *cre* boxes, which explains well the role of CcpA as a global regulator. However, the three *cre* boxes tested in that work differ very little around the middle CpG and in their symmetry (palindromic sequence) and they did not differ at the residues corresponding to our C_13_ nor A_14_.

Comparison of the high- and low-affinity *cre* boxes location in relation to the TSS also shows some trends. While the low-affinity *cre* sites can be located at any position from the TSS, the high-affinity *cre* sites cluster around the TSS, 14 and 27 base pairs upstream from TSS and 44 base pairs downstream from TSS. Simultaneously, the strongest repression by CcpA was observed for the genes with *cre* sites located around the TSS (*amyE, rbsRgmuB*) and at positions −27 (*acoRglpF*), -14 (*dctP*), +230 (*xynP*) and +372 (*treP*) base pairs from the TSS, which are separated by approximately 10 - 11-nt increments (corresponding with a full helical turn). This observation is in agreement with previous findings that activation or repression by CcpA binding to *cre* boxes is helix-face-dependent [17,45]. Also in *Lactococcus lactis* the strongest repression by CcpA was shown to occur when the center of *cre* box was located −39, -26, -16, +5 and +15 from the TSS [48].

It was shown before that genes with *cre* boxes located further upstream from −35 sequences of the promoter are subject to activation by the CcpA complex as in case of *ackA*[17], *pta*[18] and *ilvB*[19,20]. In our work however, under the tested conditions, only three genes were activated: *ilvBopuE* and *ycbP* (the two latter genes with *cre* sites predicted in this study). We did not observe activation of *ackA* in this study. This is probably due to the very low basal expression of CcpA from the TetR repressed promoter that might be high enough for binding of CcpA to the *ackA cre* box and for full activation of the *ackA* promoter. In this case, a further increase of CcpA does not result in an additional increase of *ackA* expression. Surprisingly, *pta* was downregulated. However, in this study both test and control cultures were grown in medium supplemented with glucose. The mechanism of *pta* regulation in this case is thus different from low glucose-dependent CCA. Based on our criteria, the *cre* boxes of all three activated genes are of the high affinity type. Although the *ycbP cre* box appears to be downstream to the TSS (+30), both the *cre* box and the TSS in this case are not experimentally confirmed.

Some genes and operons possess multiple *cre* boxes. Since DNA microarray technology was used in this study to assess expression fold changes of genes and operons in the presence of different amounts of CcpA, we were not always able to judge whether the effect is due to one *cre* box (and which one) or more. In our set (Table 2) there were only two operons with two *cre* boxes (the first genes of these operons are: *iolA* and *gntR*). *gntR* was weakly regulated (low-affinity *cre* box), suggesting that the regulatory effects of the two *cre* boxes do not add up to exert strong regulation. In case of the *iolA* operon, each of the two *cre* boxes is located within another gene of the operon (*cre*-1 within *iolA* and *cre*-2 within the second gene of the operon, *iolB*). In this case, the regulatory effects of these *cre* boxes could be assessed independently. Based on the fold changes of *iolA* (*cre*-1) and *iolB* (*cre*-2), both *cre*-1 and *cre*-2 seem to be of high affinity. Multiple *cre* boxes could serve for fine tuning of CcpA-regulated genes and operons.

For the genes with *cre* boxes located close to the TSS and downstream, distinct repression mechanisms were proposed. Elongation blockage (roadblock) was shown for *xylara* and *gnt* operons, as well as *sigL* and *acsA*[49-53]. Prevention of binding RNAP to the promoter sequence was demonstrated for the *acuABC* and *bglPH* operons possessing *cre* partially overlapping with the promoter region [54,55]. Transcription inhibition by direct interaction of CcpA with the σ-subunit of RNAP already bound to the promoter was shown in case of the *amyE* gene and *xyl* operon [45]. The presence of a high-affinity *cre* box in close vicinity to the TSS shown in this study, suggests that repression by inhibition of RNAP binding is one of the most effective mechanism of negative regulation by CcpA.

## Conclusions

In conclusion, we propose that besides the strongly conserved G_2_ residue and the middle CpG, the residues G_6_ and C_9_ (surrounding the middle CpG), C_13_ and A_14_ and, to a certain extent a more palindromic sequence and a location of *cre* in close vicinity to the TSS, contribute to the high affinity of CcpA for certain *cre* boxes. This finding contributes to further understanding how CcpA binding to *cre* boxes is modulated and how subregulons can be formed. However, not all the *cre* boxes behave strictly according to this rule, suggesting that *cre* affinity is possibly determined in an even more complicated way. The *cre* sequence and position may play a role simultaneously and/or more factors may be involved, for instance additional conserved sequences as shown for *ackA*[23] or sequences flanking *cre* sites as in case of *acsA*[53].

It will be interesting to use these predictions for other G-positive organisms employing CcpA, like other *Bacilli*, lactic acid bacteria, or pathogenic *Streptococci* and *Staphylococci*.

## Methods

### Bacterial strains and growth conditions

*Bacillus subtilis* strain MP902 (*trpC2*, P*tetccpA*, pWH119, Km^R^, Em^R^) was grown in rich TY medium [36] in the dark at 37 °C with shaking. The medium was supplemented with 15 μg/ml kanamycin, 2.5 μg/ml erythromycin, 1 % glucose, 0.2 % xylose and anhydrotetracycline (ATc) at different concentrations. For inoculation, synchronized stocks were used. Synchronized stocks were prepared by growing the strain in TY medium with a corresponding composition as described before [44]. At OD_600_ = 0.8, the cells were collected for determination of the CcpA production level with Western blot and for RNA isolation to be used for microarray analysis.

### Construction of the MP902 strain

All primers used in this work are listed in Table 5. To replace the *ccpA* promoter by a tetracycline-inducible promoter at the natural locus on the chromosome, the integration vector pWH849 was constructed as follows. A *ccpA* fragment truncated at the 3’ end was amplified from plasmid pWH1533 [56] using primers ccpAmut1 and Accout, restricted with *BsrG*I and *Kpn*I and cloned into vector pWH618 [56]. The resulting vector was named pWH700 and contains the terminal 246 bases of *aroA*, the intergenic region between *aroA* and *ccpA* and 689 bases of *ccpA*. Next, a kanamycin resistance cassette was amplified from plasmid pDG792 [57], using primers KmkfwR and KmkbwR, and inserted in the intergenic region between *aroA* and *ccpA* via the restriction sites *BsrG*I and *Acc*I. The resulting vector was named pWH800. The tetracycline-inducible promoter, P*tet* was amplified from the plasmid pWH1935-2 [58] with primers tetPccpAfw and tetPccpAbw. The resulting PCR fragment was used as a primer together with the primer Accout in order to fuse the tetracycline-inducible promoter P*tet* with *ccpA* at the intergenic region between *aroA* and *ccpA* in an overlap PCR with pWH800 as a template. The resulting PCR fragment was restricted with *BsrG*I and *Kpn*I and cloned into vector pWH800, resulting in pWH849. *B. subtilis* 168 [59] was transformed with pWH849, linearized with *Sca*I, to replace the *ccpA* promoter on the chromosome by the tet inducible promoter via double homologous recombination. Positive candidates were selected on TY plates with kanamycin and verified by PCR screening. The resulting strain was named MP901. Strain MP901 was transformed with pWH119 plasmid [35] carrying tetracycline repressor gene, *tetR*, under control of xylose-inducible promoter, P*xyl* (P*xyltetR*), resulting in MP902 strain.

**Table 5:** Primers used in this study

***Primer***	**Sequence (5’- 3’)**
ccpAmut1	ATAATATCTAGAACCAAGTATACGTTTTCATC
Accout	ATAATAATAGGTACCGCTTCGAGTCCGGAATC
KmkfwR	ATAATAATATGTACAGATAAACCCAGCGAACCA
KmkbwR	AATAATAATAATAGTATACTATAAAACATCAGAGTATGGA
tetPccpAfw	ATAATAATATGTACAGCATGGTCCTAATTTTTGTT
tetPccpAbw	TACTGGATACACTTATCCTTCTGCAGGCATGCAAGCTA

### Quantification of the CcpA production level with Sodium dodecyl sulfate - polyacrylamide gel electrophoresis (SDS-PAGE) and Western blotting

*B. subtilis* MP902 cells were grown in LB medium with 0.2 % xylose and 0.1 to 20 nM ATc after one overnight culture with the respective xylose and ATc concentrations. In the mid log phase, 0.5 OD_600_ equivalents of the cells were sedimented and resuspended in SBT buffer (50 mM TrisHCl, 200 mM NaCl, 10 mM β-mercaptoethanol pH 7.5). After sonification, 0.05 OD_600_ equivalents of the crude protein extracts and 200 ng wild-type CcpA purified as described previously [56] were subjected to SDS PAGE on a 10 % polyacrylamide gel. Proteins were then transferred to a PVDF membrane by electroblotting. After blocking, the membrane was incubated with a 1:10,000 dilution of rabbit polyclonal anti-CcpA antibodies [60]. For detection of CcpA on an X-ray film the membrane was incubated with anti-rabbit horseradish peroxidase conjugate and a luminol containing reagent mixture from an ECL + kit (GE Healthcare, Munich, Germany) according to manufacturer’s instructions.

To analyze the CcpA production level in the cultures used for microarray experiments, the cells were collected at an optical density of OD_600_ = 0.8 (simultaneously with collection of the cells for total RNA isolation for microarray experiments). The signal on Western blot was quantified using ImageJ gel analyzer (http://rsb.info.nih.gov/ij/). For gel loading verification, the control blots were stained with 0.1 % Ponceau S dissolved in 5 % acetic acid. Images of Ponceau S – stained membranes were obtained using GS-800 calibrated densitometer (Bio-Rad).

### DNA Microarray Analysis

16 ml of a culture was harvested at optical density of OD_600_ = 0.8 by centrifugation at 8,000 × g for 2 min. The pellet was rapidly frozen in liquid nitrogen and stored at −80 °C until RNA isolation. DNA microarray experiments were performed in general as described before [44]. Total RNA was isolated using High Pure RNA Isolation Kit (Roche) according to the manufacturer’s protocol. RNA quantity and quality were tested with a ND-1000 spectrophotometer (NanoDrop Technologies) and an Agilent Bioanalyzer 2100 with RNA 6000 LabChips (Agilent Technologies Netherlands BV), respectively. The amino allyl modified cDNA was synthesized with the Superscript III Reverse Transcriptase kit (Invitrogen), purified with Cyscribe GFX purification kit (Amersham Biosciences), labeled with Cy3 and Cy5 dyes and purified again. The labeled cDNA was hybridized to oligonucleotide microarrays in Ambion Slidehyb #1 buffer (Ambion Europe Ltd). Slides were washed, dried by centrifugation and scanned with a GeneTac LS V confocal laser scanner (Genomic Solutions Ltd). Scans were analyzed with ArrayPro 4.5 (Media Cybernetics Inc., Silver Spring, Md., USA). The resulting expression levels were normalized with Micro-Prep [61] and subjected to a t-test using the Cyber-T tool [62]. All microarray experiments were performed in three biological replicates. The complete microarray data is available at the GEO repository (http://www.ncbi.nlm.nih.gov/geo/) under accession number GSE35154.

The sequences of the *cre* boxes known from DBTBS [41] were used to generate a weight matrix in Genome2D [43]. The resulting Weight Matrix was fed into the Genome2D [43] to find the potential *cre* boxes in the whole genome of *B. subtilis*. In this search, a cut-off of 8.96 was used. The promoters (−35 and −10 boxes) and transcriptional start sites (TSS) were predicted using PePPER (Prediction of Prokaryote Promoter Elements and Regulons) tool [63] and sequence analysis. For the annotation, GenBank file NC000964.gbk last modified on the 19^th^ of October 2011 available at http://www.ncbi.nlm.nih.gov/guide/ was used. The operons from DBTBS database [41] were confirmed with experimental evidence from microarray results obtained in this study (clustered up- or downregulation of genes belonging to one operon).

## Abbreviations

*cre*, Catabolite responsive elements;ATc, Anhydrotetracycline;TSS, Transcriptional start site;G6P, Glucose-6-phosphate;FBP, Fructose-1,6-bisphosphate;RNAP, RNA polymerase.

## Competing interests

The authors declare that they have no competing interests.

## Authors’ contribution

BCM, WH and OPK contributed to the design of the study. OPK and WH conceived the study. GS, RD and MP constructed the MP901 and MP902 strains. MP and RD performed Western blot experiments. BCM, MP and AdJ were involved in analysis of the data. BCM and OPK were involved in interpretation of data and writing the manuscript. All authors read and approved the final manuscript. OPK gave final approval of the version to be published.

## Authors’ information

The authors wish to dedicate this work to the memory of Prof. Wolfgang Hillen who died unexpectedly on 17 October 2010.

## Supplementary Material

Additional file 1 **Gene expression fold changes "CcpA induction" vs "no CcpA induction".** Expression fold changes in three microarrays experiments with low, medium and high CcpA expression induction. Activated genes - positive values, repressed genes - negative values. Click here for file

Additional file 2 *** Cre* boxes predicted in the* Bacillus subtilis* genome using Genome2D software and 8.96 calculated cutoff.** Click here for file

Additional file 3 ***Cre* boxes predicted with Genome2D software using cutoff 8.96 and found in upper and lower strand within −500 and +100 nucleotides from the start codon of the first gene of operon.** Click here for file

Additional file 4 **List of significantly regulated first genes of operons (1.8 < fold change < −1.8) with *cre* boxes within −500 and +100 nucleotides from start codon. **Significant fold changes are shown in bold. *cre* box to start codon and transcriptional start site (TSS) distances are calculated from conserved G residue in the middle of *cre* box to first nucleotide of start codon and to TSS, respectively. For *cre* box affinity determination criteria see main text. Click here for file

Additional file 5 **First genes of operons with *cre* sites.** Start codon and further coding sequences are shown in lowercase and intergenic regions in uppercase. Underlined – predicted *cre* boxes, bold – *cre* boxes known from the literature, solid box – promoter known from the literature, dotted box – predicted promoter, dark grey shadow - transcriptional start site (TSS) known from the literature, light grey shadow – predicted TSS. Click here for file
