# Supplementary material for: High- and low-affinity cre boxes for CcpA binding in Bacillus subtilis revealed by genome-wide analysis
Source: BMC Genomics. 2012 Aug 17;13:401. doi: 10.1186/1471-2164-13-401 (PMC3463425; doi:10.1186/1471-2164-13-401)
Supplement: Additional file 5 — First genes of operons with cre sites. Start codon and further coding sequences are shown in lowercase and intergenic regions in uppercase. Underlined – predicted cre boxes, bold – cre boxes known from the literature, solid box – promoter known from the literature, dotted box – predicted promoter, dark grey shadow - transcriptional start site (TSS) known from the literature, light grey shadow – predicted TSS. [file 1471-2164-13-401-S5.pdf]

Additional file 5. Regulated first genes of operons with *cre* sites. Start codon and further coding sequences are shown in lowercase and intergenic regions in uppercase. Underlined – predicted *cre* boxes, bold – *cre* boxes known from the literature, solid box – promoter known from the literature, dotted box – predicted promoter, dark grey shadow - transcriptional start site (TSS) known from the literature, light grey shadow – predicted TSS.

Gene	Upstream sequence	*cre* to TSS distance [a]	TSS determination reference
abnA	ACGCGGTAAAGTTAATTACAGAAGTCATTAAGAAGTTAGACCGAAAAACGGTTGACGAAATTACGTACCAATAAAGATACGGAAGAATCCGCCTATTGAGGTGGATTCTATTT<br>TTTTGTCTGTACAAATTACAGCATAGTGACTACAATAAAGGGGATACCGAAAATTTCCTGAACATGACGATATACGATACGCAGTCGATTTGACAGGAAGGGAGGATAAATAA<br>TCTAATTTGTAAGC*G*CTTTCTAAAATAAAGGAGGTTGAACAAAAttg…	+85	[1]
acoA	CTGATTTTACGGGCTCAAAAGACTGGCACACTTCTTGCATTTATAATGGTGAACCCTAAATAGAAGGAGGCGCACAAAatgaaattgttaaaacgagaaggcttgtcattaactgaggaaaaa<br>gcgctgtggatgtaccaaaagatgctggagatcaggggctttgaagacaaagtgcatgaactgttcgcccagggagtgcttcccggattcgttcatttatatgccggtgaggaagccgtggctgtaggggtgtgcgctcatttacatg<br>atggcgacagcattacaagcacccacaggggacatggacattgtatcgccaaaggctgtgacctggacggcatgatggcggaaattttcgggaaagcgaccggattgtgcaaaggcaagggcggttctatgcacattgcggatctt<br>gataaaggcatgttaggcgcaaatggaatcgtcgggggcggctttacgctcgcatgcggatcagcgctcacggctaaatataaacagactaaaaa**tgtaagc*g*tttgct**tttcggggacggggcaaataaccaaggtacc…	+462	[2]
acoR	TTGGCCCGGATGTAACCGAGCTCATCGGCCAAGCGGCAGCGATCATGAATGGTGAGATGACGGCAGATATGGCGGAGCATTTTATCGCCGCCCATCCGACTTTATCGGAAACA<br>TTGCATGAGGCGCTGTTAAGCACGATCGGCCTTGCGGTACATGCATAATAAAGGAAAAAGCAGGCGCATGGATATAAGGCGCCTGCTTTTTTATTGT**TGAAAGC*G*CTTTAT**TTT<br>TCCCCTACAATAGATGAAAACGGCGTGTAAGGGAGGAGCGATCAAGGAAatg…	-27	this study
acsA	TTTATGAAACTCATAACCTGCCAAATCCTCTGGAGAGACAGGCCCTTCTATCAGTAAAGAGCCGGTTGCTGTTTTGATGTTTGCTGAATGGTATGTTTTATGATGTTCCACTAATT<br>CACCGTCCCATCAATTCTTATCAAAAATACATGCCTTCTTTAAGAATACCAAATTATAAAGCGTTTTCAACATGGTTTATATTTTAAAAATTGAGAAGAATATGAATATATACTAT<br>AATAATTGTGACAACTTCAGCAAAGGGGGATGTTTGGGCatgaact**tgaaagc*g*ttacca**gcaataga…	+44	[3]
acuA	AGAGAAATGTTTCTCTGCCTCGGCCCAATCAAAATGCCGGTACGTTTCTTCATAGTTTTTTAAGTTATGATCCCCCTCTATTGCTGGTAACGCTTTCAAGTTCATGCCCAAACATCC<br>CCCTTTGCTGAAGTTGTCACAATTATTATAGTATATATTCATATTCTTCTCAATTTTTAAAATATAAACCATGT**TGAAAAC*G*CTTTAT**AATTTGGTATTCTTAAAGAAGGCATGTAT<br>TTTTGATAAGAATTGATGGGACGGTGAATTAgtg…	-26	[3]
amyE	CGGTAAAAGAAGAAATAAAAAAGAAATCATCTTTTTTGTTTGGAAAGCGAGGGAAGCGTTCACAGTTTCGGGCAGCTTTTTTTATAGGAACATTGATTTGTATTCACTCTGCCA<br>AGTTGTTTTGATAGAGTGATTGTGATAATTTTAAA**TGTAAGC*G*TTAACA**AAATTCTCCAGTCTTCACATCGGTTTGAAAGGAGGAAGCGGAAGAATGAAGTAAGAGGGATTTT<br>TGACTCCGAAGTAAGTCTTCAAAAAATCAAATAAGGAGTGTCAAGAatg…	+4	[4]
bglP	AACGATTCATAACCTTGATTGGAAAAAATGCCTTTCAAGGTTTTTAATTACAATAATTTTTTATTGAAGTTAATACTAAGTTCAAATCTTTATGATTACTTTTGTTCTAGAAACGAT<br>AGTCAACACCAAAAA**TGAAAGC*G*TTGACA**TCTCACGAATCTAGTGCTAAAGTATACCTACAAACAAATAAAACGACCCGTCAGGATTGTTACTGCGAAAGCAGGCAAAACCTA<br>AATGGTGCAGGTGCAGGAGGAGACTCTCGCAATCTGTGTCATTTAGGTTTTTTTATTCTCTTTTAGGAGGTGATGCAGCATTTGATACAAAAAAAGCTGGTGAAAGGCGGCCAC<br>CTTTCATCCAGCAGTCCCCCCATAAAAAGGAGGTATACACatg…	-36	[5]
cccA	GTTGCGGAAACTAAGACATCCTAGCAGAAGTAAACGTTTGAAAGATTTCCTTGAATAAGATGGAACGGGTCTTGAAGATCCGTTCTTCTTTTTTTAAAAAGATATATGGATAAT<br>ATGCCTTTATTTTACTGAAAAATGATGTCATTTGCAAATGAACATTGTGGTGAAAAATTTCAAAATCTAATTCCATATTTTCTATTGTAAGC*G*TATACAATACATTATACAATAGA<br>ATAAAAAGGATATTAGAGATTTAGGCATGTTTCTATTCAATTTTGTATAAAGTGTGAATAAAAAACTTTTGTATAGCAATCCATTTACTTTTTGTAAAAATAAGTTAGAATTAGAA<br>GTGTTTACATAGGGGGAAGGATCAAAAAGGGGGAAGGGAAatg…	-29	[6]
citM	TTATTTAAACAGCAGGGAAACGAAACGGTTTTTAGAAGAGCAATCTGCTTTTTATCAAAGCATCATGACAGGAAATTAAAATCCCGACATCCCGGGATTTTTTTCATGCCGAAAA<br>TTGATCAAAAAGAACAAAACGGTTTTAAAAAATTAAAAATACAAAAAAACCAAATTATTTACTTGCGGTTGGTTTTCCCATACGATGGCAAAAAGGCAAGACAAAAAGGGGAG<br>TAAGGGAGAAAAAAA**TGTAAGC*G*GATTCA**TTTAAGGGGGAATGGATgtg…	+46	[7]
citZ	GAGCGGGGATTGCTTATTTAGCCATGCAGATCGTAAAACTGTTCACTTCATAAAAAAAAGCGGCCTTAAAGCCGCTTTTTTTTATGCAAAAACCCGCAAATAAAGGCGTTTTTTC<br>GGGGAAATCACAGAATGGAAAAAAAAAAATTATAGGTAAACATTTAACAAATGTCTGATTATTGTTTATAATGAGAATAGGCTTAAACTTAAATAAGCTTATAAAAATTTGTTAT<br>GTTCTTTCAAAAGCAAAGGGTTTTAGTTCCAGCAGCCAGTAAAA**TGTAAGC*A*TTTTCT**TTTTGGGGGAGAGAAATACTTGCATCGTGCTTTTTAAGTAAGCCTCATGTTTTTACA<br>ACACTCTTAAAGGGGAAATTTATTGAAAAGGAGATGTTATATatg…	+88	[8]

csbX	GCTTTGGTGAATCCTTCTTTTGCGGAGCTGGGTGATATTGATACAGCTGTTATTACACTGACTTTTGAAAACGGTGCCATGGCTGTGATTGATAACAGCCGCCAAGCTGTGTACG<br>GATATGACCAGCGGGTTGAGGTTTTCGGGACGAAAGGCTCTGCAGCGGCTGACAATAGCAGGCCGACAACGGTTGAGGTGTCAACAGCTGATTTTGTGATGAAGGATAAACC<br>GCATTTCTTTTTCCTTGAACGCTATAAGGATTCATATGAGGAGGAGATTCTCCGTTTTGCAGAAGCGATCGGCACAAACCAGGAGACTCCCTGCACCGGCAATGACGGTTTACA<br>GGCCGGGAGGATCGCCAGAGCAGCACAGCAATCGCTTGCTTTTGGCATGCCTGTTAGCATTGAGCACACTGAAAAAATCGCTTTTTAATCTAACAGGATTACAATTCAGCAAGC<br>TTGGGTATATACTCCATTGATACTTTAAGTAGGCGGTGGAGAAAatg…	-401	[9]
cstA	ATAACAGAAAAAATGTCGTTCCGCATTCCAACGGATCATCGTCTGTCAGCGAAAACGGCTTAACTGCTCATTTCACGCCGCTTTCCTGGAATGTGATCCGCCTGAAAAAACAGTC<br>ATAAGAATAGCAAAGCCGGAGATTTCTCTCCGGCTTGTCTTTCAACTGCCACGAGCCGGCCCATTCCAGCCGGCTTTTTGTATAGGAAAAAATGACCGCTTTTCACCATGAAATT<br>ATGATATATTTATGAAAAACAGAAAAGGGGATGGGAGAAAatgaatgcggttacaattgtgatagcatcaatgtgtattttggctat…	+32	this study
cydA	TCGAGTGCTGGGGCGGGGGCAAGCATTTTGTTCGCAAGGAACGATGTGTTGATGTACGATAATAAAGGGTCATTTATCCTAGCAGCCGACATAAATAAGCAACAAAATAGACA<br>AAAATCCGTCACATAGTGCGGGACTTTTTAGGTATTTTAGGCTTTATTTT**TGAAATGAATCGTT**GTAAAGTACTTAATATGAACCAGTCAGAGATTGTGTCATTTGGTCAGTCTG<br>GCAATCTTGCATCATATTGGATGACTTTTTGACACATTTGTGAAATATTGAGCAATATTTTTTTCGTCTATTTTGTGAATTACTGATCAAAGTCTCGGTTCTATTTGTGAAGTAGTG<br>AGCAAATTAACAGTTTTAACCGGAAATGGAGGAGAAAGCatg…	-21	[10]
dctP	CCTTTCTGCTGAAGAGGTTGCCAAGGCGCTCGGCATCGCCAGAGTGACAGCCAGACGATATCTTGATTACTTAGAAAAAACCGGGATCATCAAATTGGACGTTCAATACGGCG<br>GAGTCGGCCGGCCGGTCAACCGCTACGTGTTAAAAGGATAAACCTGAGACCAAAAAGACCAAAATGTCCGTTATGATCATAAACTTCCCCAGACTGTA**TGAAAACGCTATCA**T<br>TCTAGTAAGAAAGACACAAAAGGCGAATAGGACAGGGGGATGAAGGATatg…	-14	[11]
drm	AGCGGTGCAGGAAATGCTCGGTCATGCGGATATATCCACGACGCAAATTTATACGCATGTGACGAAAACAAGGCTGAAGGATGTGTACAAGCAGTTTCATCCGCGAGCATAG<br>CCGCAGGTGGCTGCGGCTTTTTTGTTTAGAGCGACAGAAAATATTGAAGTTTCTCATTATTTCCAAATACATTGTCAACGGGAA**TGAAAACGGTTTAT**ACTTGAGTTGTCAGAC<br>CTCTTAGCGATACAATAGACGTATAGAAAAAAAGGAGGCTTTCAAGatg…	-16	[12]
glpF	AGCCTGACTTCATCGAAGTGCTTCCCGGCATCGTTCCGTCACTCATTCAGGAAATAAAAGAGAAAACAGGGATTCCCATCTTTGCCGGGGGTTTCATCCGTACGGAAGAGGATG<br>TAGAGCAGGCATTGAAAGCGGGGGCTGTAGCTGTCACAACATCTAATACCAAATTGTGGAAAAAAATATGAAAACTTTTTGACGGAAAGTGAT**TGACACCGCTTTCA**TGCACTG<br>ATACAATTGCACTAGGTTAATACATTGTGATGGAGAACTCGGAGACCACAGCAGCTCTTTACGGCAAATGTTTATGCACCCGTAAAGCGGTTTGTTGTGGTTTTTTTATTCTCTT<br>CTTCTCTATCATGCTTTTTAATCGTGACTTTAGGAGGAATGTGCTatg…	-27	[13]
gntR	TCTTTCTATCGCTTCGGCTGCCTCCAGAGCTGATAAGCTTTCCTTAAAAGAATCAGGTGCAATGATGATTTTCATCCAAATCGCCTCTTTCAAAACATGATTCACTTAACCTATTGA<br>TCTCCAATGTACCATAATTGATCTGGAAATACATACCATGCAATATGGTAAAAATTTAAATAAAAATTAGAAA**TGAAAGTGTTTGCA**TAAAAGAAATATTCACGTTATCATACTT<br>GTATACAAGTATACTCCTTGAGTGAGGAAGGTGAGTGTatgctagactccaaagacctgttgtatcccgcaaaatggctctcaaaagcgtcaaccggagttcgtgtcgcatacgagctgagaatgcggatcgtttc<br>aggtctgat**tgaaagcggtacca**ttttatc…	*cre1*: -41<br>*cre2*: +148	[14]
hutP	ATTGAGAGTTAGACAGCGGCGCTTGCCTCCTTGTCTGGATATCGTGTGCAATCGCTGAAAAAACCTGTTCTGCTTTCTGCGTCACGCTATTACAATAGCAATCTAACTTGTTAAA<br>GACTATAAAAAAACCTTTTGACTTCTGCTGCTGAACCAATTAATATAATACTCAGTTAATAGTTATCAGAATTTTTAGGAATTGAgtgattcatatgacactgcataaagagcgtcggatcggc<br>cggctgtctgttctcctgctgctgaatgaggcggaagaaagtacgcaggttgaggagctggagcgagacggatggaaggtctgtcttggcaaggtaggatcaatggacgcacataaagtagtagccgcaat**tgaaaccgcttcca**a<br>aaagagc…	+209	[15]
ilvB	TGCAATTCCTTTTGTACCAATAA**TGAAAGCGTATACA**ATATAGATTGATTAATCAAAATTGTCTAATAATTTTAAAAAATGCTGTTGACACTGCGTCCAAAGCGGCGTAATATGA<br>GTTCAACAAAAGATAAATGCAAGCTTCACAAGCGAAAATCATCGCAGTATGATTCTAAAAAAATGAAAAACAAACGACCTTCTTGAACAGCTGGAAAGCCGTTCCGAAGGCTG<br>AATATGAAAAGCGCAGATGAGGATAAGTAGCCTTGATAAAGTTTTCCACAGAGAACCGGGTTAGCTGAGAACCGGCGAAGCTTTACAAGGTGAACTCGCCTCAGAGTGCCAG<br>TCTGAAATGACAGTAGGACTTGGCCGGGTGAACTTGATTCACTCGTTACTAAAGCGGATAGAAATATCCATGAGACGGCCGATTAACAGGCCGTAAACAAGGGTGGTACCGC<br>GGAAAGAAAAGCCTTTTCGCCCCTTTTAGCTATCGCAGTTACTGCGCGGCTGATTGTGGGCGGAAGGGCTTTTTTTATTGAATAATCAGCTATCTAGCTAATGAAAAGATGATCT<br>TTAAAGGATGAAAATCCAAAAGGAGGAACTAAAatg…	-88	[16]
kdgR[b]	ATTAAAAAAACTCTTCCCAAATTTTAAAATTATGGTATTCTTTTGTTGTAACGGTTTTGAAATCGGTTTCAAATATATGAGATACAGGAGCACTTGAatgaaaaagaaaacaacaggcc<br>atacaaccatcaaagacgtagctgaatgtgcaggagtttcgaaatcgacagtttcccgctatattaacggaaaaattgacgcgatttctcctgaaaaagtgaaaaacattaaaaaggcaatcgcagaattgaattatcggccgagta<br>aaatggcacaaggtttgaaaataaaaaaaagcaagttaatcggatttgttgtggctgacattacaaatcctttttcggtcgctgctttcaggggagtcgaagaggtttgtgatcaatatggatacagcattatggtgtgcaacacagat<br>aacagtcctgaaaaagagcgggaaatgctcttaaagctagaagcccattcggttgagggcttgattttaaacgcaacaggggaaaacaaggatgtactccgcgcttttgctgaacagcaaattccaacaatcttaattgaccggaa<br>actgccggatctgaaactggataccgtaacgactgacaacagatggatcacaaaagagattcttcagaaggtgtacagcaaagggtatactgatgtcgcgctgtttaccgaaccgatttcctctatcagccctcgtgcagaaagagc	+2445	[17]

	agctgtgtatcaggaaatggcttcggttcaaaatgtgaacgggcttgtcagattgcatgaaattgacgtaaaggacaaggagcagcttaaggcggagcttcgttcttttcacaaggaaatgccggagcaaaaaaaagcgattctggc<br>tttgaatggcttgattatgctgaaaatcatcagctgtatggaagagcttggcctgcgcatccctcaagatatagggattgcaggattcgatgatacggagtggtacaaactgatcggtccaggcattacaacgatcgcccagccgtca<br>catgatatgggcaggacggcgatggaacgtgtattaaaaaggattgaaggtgacaaaggagcgcctcaaaccattgaattagaggcaaaagtcatcatgagaaagtcacttTAATCCTAGGTGCCCTCAACTAAGAG<br>GAGGTTGGGAAGAGatgaagcttgatgcggtgacattcggggaatcaatggccatgttttatgcaaatgagtatggaggccttcatgaagtatccactttttctaaagggcttgccggagcagaaagcaatgtcgcctgcggc<br>cttgccagactcggatttcgaatgggatggatgagcaaggtcggcaatgatcagctcggaacgtttattttacaagagcttaaaaaagagggagtggatgtgtcccgcgtgatccgctcgcaggacgaaaatcccaccgggctgctg<br>ctgaagtcaaaagtgaaagaaggcgacccgcaagttacctactataggaaaaactcagctgcaagcacattaactacagctgaatatccgagagattattttcaatgcgcaggccatttgcatgtgacaggtattccgcctgctttat<br>cagccgagatgaaagacttcacgtatcatgtcatgaacgacatgagaaacgcagggaagaccatttcattcgaccctaatgtaagaccttcgctttggcctgatcaagcaacaatggtacacacaatcaatgatctggctgggcttg<br>cagactggtttttcccgggtatcgcagagggagagctattgaccggagaaaaaacacctgaaggcattgccgactattatttgaaaaaaggtgccagctttgttgctattaaacttggaaaagaaggcgcttactttaaaacgggga<br>caagtgaaggatttctagaaggctgccgggtcgaccgggtggttgatacggtcggcgccggagacggatttgcagtcggtgtaatcagcggtatcctagacggtttgtcgtacaaggatgcggtgcaaaggggaaatgcgattggc<br>gctttgcaagtgcaggcaccgggggatatggacggtttgccgaccagggagaaattagcttctttttttatctgctcaaagaacggttcaccaaaagaaaggggattatTAATatggagtccaaagtcgttgaaaaccgtctgaaag<br>aagcaaagctgattgcagtcattcgttcaaaggataagcaggaggcctgtcagcagattgagagtttattagataaagggattcgtgcagttgaagtgacgtatacgaccccccggggcatcagatattatcgaatccttccgtaatag<br>ggaagatattttaattggcgcgggtacggtcatcagcgcgcagcaagctggggaagctgctaaggctggcgcgcagtttattgtcagtccgggtttttcagctgatcttgctgaacatctatcttttgtaaagacacattatatccccgg<br>cgtcttgactccgagcgaaatta**tggaagc*g*ctgaca**ttcggttttacgac…		
lcfA	TACCTTTCTTTTTCTTTTTTTTGCCGTCCATCTGCTATAATGATAAGGTGGATCTCTAAAATCGAAAGGGGAGGTTTTatgcagtctcaaaagccgtggcttgccgagtatcccaacgatatcccgc<br>atgagcttccgttgccgaataaaaccctgcaatccatcctgacagactccgccgcacgattccctgataaaaccgcaatatcgttttatggaaaaaaactcacctttcatgacattctgacggatgctcttaaacttgcagctttttttgca<br>gtgcaatggcctgcaaaaaggagacagagtggctgttatgctgcccaattgcccgcaaacagtgatttcttattatggcgtcttgtttgccggtggcatcgtggtgcagacgaatccgctttataccgagcatgagcttgaataccagc<br>ttagagatgctcaggtaagtgtaatcatcaccttagatttgcttttcccgaaggcaataaaaa**tgaaaac*g*ttatca**atagtcgaccagattttgataaccagtgtaaaagactatttgccttttccgaaa…	+450	[18]
levD	AGCTCGGACTTCGCATAAGTGATGATGAAAAATTATTTATAGCCGCCATATTTGCTGAAGAAGTGCATGGCCAGCTGTTTTAAAAACAGTTTTTTCATATGAACCTGTATTAAAT<br>GGAACACCATTTTAATACAGGTTTATTTTTTTCGTTTTAAGTGTTTCAACAACAAATTGCTATTGGCTGAAATAACAA**TGAAAAC*G*CTTAAC**ACAACTGTGTTGGCACGATCCTT<br>GCATTATATATGGATGTACAAAACAGGAAAGGAGCAATAGATatg…	-45	[19]
malA	CGGCATGTATCCGAATCGTACAAAAGAACCTTTTCATAAGAATTGGAAGGGCGTATATTCACTTAAAATTCACAGTTGGTGAGACTTTAAGATTACAAAAAAGGTAAAAAAACC<br>AAATCTCTCAGACATAAGGCAAATGAGAAATTTCCCGCTCTATGGGAAAAAACACTAAAGTTGATCAAATGACCTAAGTGCGCCAAACGTGTTACGGGACGAGCTATCTCATG<br>GTATAAATGGAAT**TGTAAAC*G*TTATCA**AGGAGGTCGTCATatg…	+6	[20]
manR	TCTTCATCATGTTCTTACTTTCGTACTCATACATTAGAAAATCTTCGTTCTAAATAGGTGAAGAATTGGAGTATTTTTATGAATTTCTGCTGAATATACATTACATAGCAAACTCAA<br>AGAGTATAAAAATCGCTTTTTTCCGGAAGCTTCGGTAAAAAACGAAACTTTTGTCTCTATGATTTTGTTTTATAATGTAAAC*G*GTTTCTTATATAGTATACTTATACTATCAATTTG<br>CTCAAGTAGATACTGACAGGAAGGATAGAAAAACAGatg…	0	this study
mleN	ATGACAGGGCAATCTGTCCGGGATCTTTGTCTGCAGCATGATGTGCTGACTGAAGAAGAATTGGATATTATTTTAAACCCATATGAGATGACCAAACCAGGTATCGCAGGGAA<br>AGAACTATTAGAAAAATAAAATTTGTATAAAATACAGCTTATTCATCTCCGAATAGTCGGATGAATAAGCTGTTTACAACATAGGGATGAGGAAGTATAGTATACGTGTTGCAC<br>TATATATTGAAAGC*G*TTTTAGCTTTTACATGAAGGGGGACTTCTTttg…	+21	this study
iolA	ATTAATATAATAAAAATAATGAAAAAATGTAGTGTTTATGATTGACTTATGGGTATTATGCGATTAGAATATAACCAAGAAATGACCAAAAAGTAACCTGTTGTGCATGAAATG<br>GTTGCTTGTATGTAAATATGCATCCGCCATTTATTTTTTTGGTGTTTT**TGAAAGC*G*TTTAAT**TCTTGGCTTGCTGAAAAGTGGATATTGATATGAATCTATCCGGAACCATCCATA<br>CAAGTGTATGTGTTTACTAATGAAGGAGGCAATAAGAatggcagaaatcagaaaattaaaaaactacatcaacggtgaatgggttgaaagcaaaacagatcaatatgaagatgtcgtcaatccggcgacgaaa<br>gaagtgctatgccaagtgccgatttctacaaaagaggatattgattacgcagcgcaaacagcggctgaagcatttaaaacatggtcaaaggtggcggttcctcgccgcgccagaattctatttaacttccagcagctgctgtctcagc<br>ataaagaagaacttgctcatctaattaccattgaaaacggaaaaaacacgaaagaagctttaggtgaagtgggacgcgggattgaaaacgtagagttcgccgctggagcaccttccctgatgatgggtgactcacttgcttccatcg<br>caacggacgttgaagcggcgaactaccgttacccaatcggagttgtcggcggaatcgcgccattcaacttcccgatgatggttccttgctggatgttcccgatggcgatcgcgctcggcaacacatttattttaaagccatctgagcgg<br>actccgctgttaacagagaaattggttgagctttttgaaaaagcgggccttccgaaaggggtgttcaacgtcgtatacggtgcgcatgacgttgtgaacggcatcctcgagcatcccgaaattaaagcgatttcatttgtaggctccaa<br>gccagtaggcgaatacgtctacaaaaaaggaagcgaaaacttaaaacgcgttcaatctctgactggcgcgaaaaaccatacgattgtcttgaacgatgcgaatcttgaagacacggttacaaacatcgttggagctgccttcggttc<br>tgccggtgaacgctgcatggcttgtgcggttgtgacagttgaagaaggaatcgccgatgaatttatggcgaagctgcaggaaaaagtggcggacattaaaatcggtaacggccttgatgacggcgtgttcttaggaccggtgattcg<br>cgaagacaacaagaagcgcacgctcagctatatcgaaaaaggtcttgaagaaggtgccagactcgtatgtgacggacgtgaaaatgtgtctgacgacggctactttgtcggcccgacgatctttgacaatgtcacaacagaaatga<br>cgatctggaaagatgaaattttcgctccggtcttatctgtcatccgtgtgaaaaacctgaaagaagcgattgaaatcgccaataagtctgagtttgcgaacggcgcctgcttgttcacatccaactcaaacgcaatccgctacttccgt<br>gaaaacattgatgcgggaatgctaggcatcaaccttggtgtgccggctccaatggcgttcttcccattctcaggctggaaatcttcattctttggaacgctgcatgcgaacggaaaagacagcgtcgacttctatacacgtaaaaaagt<br>ggtgacggcaagatatccggcacctgatttcaacTAAACGACGAACAGCCGAACGGCGTCCATGTGCGCTGTTCGGACTCATTCTGCCAATAGAAAGAGGAGGGCTTCTCTatgagttatttgtt<br>gcgtaagccgcagtcgcatgaagtgtctaatggggtcaaactcgtgcacgaagtaacgacatccaactctgatctcacttatgtagagtttaaagtgttagatcttgcatcaggttcaagctatacagaagaattgaaaaaacaaga<br>aatctgtattgtggcggtaacggggaaaattacagtgacagatcatgagtcgacttttgagaatatcggcacgcgcgaaagctattttgaacgaaaaccgacagacagcgtctatatttcaaatgaccgtgcatttgagatcacagc	*cre1*: +93<br>*cre2*: +2404	[21]

	ggtcagcgacgcaagagtggcgctttgctattctccatcggaaaagcagcttccgacaaagctgatcaaagcggaagacaacggaattgagcatcgcgggcaattttcaaacaaacgtactgttcataacattcttccggattcaga<br>cccttcagctaacagtctattagtagttgaagtctatacagacagcggcaactggtccagctacccgcctcacaaacatgaccaagacaacttgccggaagaatctttcttagaagaaacgtactaccatgagttagacccgggaca<br>gggctttgtgtttcagcgcgtatacacagatgaccgttctattgacgagacaatgactgtgggaaa**tgaaaac*g*ttgtca**tcgttcctgcgggataccacccggtaggcgttccggacggatacacatcctactatttaa…		
msmR	AGGCAAATCACCAAGCAATCGAAACAGTTGCTTTCTTCTTTCGGCTCTCATCCCATCACCCTTCCTTATTCGCTTACTTCACATTTACGAGACAGATCTGGGTTCCCCTTTATTTTTC<br>CAACATAAATCACATCATTTACTAAATATTTAGTAAATATTATGGAATATTTCGAAAACAATTATTTGTAACC*G*CTTACTTTTATATGATAATATCAATTTATCAAAAACAGATGAG<br>TTAATATTTTACTAAATAGATGAGAGGGATACCCatg…	-28	this study
msmX	CACATTAACAGCGAAAAAGCTGCATCTGCATCGCAACAGCCTGCAATATCGGATTGATAAATTTATTGAGCGGTCAGGGATAGATATCAAATCGTACAAAGGCGCGCTTCTTGC<br>CTATTTTATCTGCCTGCAAAATGAAAGTTCAGAATAAATTGGAAATATGCACGAAAAATGGCTGGGTGATTTTATGAACGCTGCCCATTTTAGCGAGAGGGAATTGCTGTTACA<br>CTCAATATA**AGAAAGC*G*TTTACA**ATAACAAAGGGGGATGTTAGatg…	-15	this study
mtlR	TCCGCCGCGATAAATGCGTTGTCATACAAGCTTGACCAGGATCAATGGTTCCGCATTTTTACAGCCGTACAGGGTTACGATATTCCATAAAAAGCATCAGTTTACCAGCTGATGC<br>TTTTTCAATATTGTCACACCGCTTAGTGCCAAATTACCATTTATAAGTGATTGATTCTTTCTTGAAAGC*G*TTTTATATTAAAAGGAAACCTCTCTATATCCTCTACTGCATCTCCTT<br>GCGACTTCATCCGCACATCAAGATTGGCTAGGAGGTCTTTTTTatg…	-16	this study
odhA	TGTATCCGCTGCTGAAAAAACTGCTTCATAAAAGCATAGGATAGCCCTTAATCCTATGCTTTTTGGCGTTTGTTTTTTCGAATGATTAAATTTTTTGTTTTTTATAAAGGTTTTTTAC<br>TATTTTGTGAACAATCAAGGTAGAATCAAATTGCAAACAGTGGTAAAATATCGTTGGAAGC*G*TTTTTATTACATAATTTTGGACACAGCGTTCGAAAGTTATTTCAAGATAGTG<br>AATCAATCACTTGATTATTTAAGTTGGGGGTAATATTCAAatg…	+21	[22]
opuE	TCATATAATACTGATCATATCAAAAACAAGCCCTTTAAAGCAACAAAACGCCTTGAGACAGGCCTGTGTTTTCGAAACAATACCCTATATATTTTATACTAAAATGAGGAGAAGG<br>AGAAACGAAATATTTTTCTGAATATTTTTACTTTAATTATTTTTTGAAAGC*G*TTTTATTCTTTATTCCCTTCTAGGTTTGCAACGGTATTTCCAGCTTATGATCAATGTTTCATCGCT<br>TGAATGGCCGGGAATACTTTGTTAGGTTAAGTTACCAAGTGCACCCGCAAAAGAATTTCTTTTGTTTTGCACTTACGCCAGGCTGCTTAACTGGCACCATCAATTTGAGAGGTG<br>ATGCAACATTATTAGAAATGAAGAGAGGGTAAAACgtg…	-103	[23]
pbuG	GGAATAAGAATCAATTAATGGAAACCATCTTTTTGGCAATTTTGCCGGGAAGATGGTTTTATTTGTTTATTACGAACAAAATCCGATTTTGCGCGACTATTGTTCGCTTTTTGTAT<br>TGTTTTTTCAAAATGAACTTGGTACAATAGACACAGAAATCAAATAAGATGAATTCGTATAATCGCGGGAATATGGCTCGCAAGTCTCTACCAAGCTACCGTAAATGGCTTGAC<br>TACGTAAACATTTCTTTCGTTTGATATAAATAAAACACGGTTATTTATTCAAACTGAAATCCGTCTGTAGTCAAGCGTCCCAAAATGTATTGGGACGTTTTTTATTTGGCGTTTTCA<br>GGACAGAATAAATAGCAAAGAGTGAAGGGAGTCAAATAGCttgaaaac*g*tttttt cagtttgatgagctgggcaccagctat…	+245	this study
pta	TATCTTCACTCCCATTATCTTTTCATGATTGCCTTCACTATACCATAGTTTATCCGATTCTACTGTGGAGAAACCTTGAACATCCCTTCCGTATAACTCCATCAAAATAGAAGAACCT<br>ATAAGGGAAGATTGAGTCACTTAAATTAGTTAGAAAAATAAGATTTTTTTA**TGAAAGC*G*CTATAA**TGAAAGTTGGCTGTTTGAATTTGATTGGAAGAAGAGTATGCTAGTAAA<br>AGAAAGCGTTTTTGTAACTTTTTGAGGAGGTTTTATTgtg…	-55	[24]
rbsR	TTTTCTGGGATGATAGTCTTTTCTGTTTCTCCCCATTTACAGGTCTAAACGCATGACTTTGAAACAATTTTAATAAAACTTAATATTTGTTCAAGAAATCTTCATCCATATTTGTGAA<br>GACTTTGTCAAAAAAAGAGTGAAAACCTTAAATTTTTCAATTATATATACAATTTACAATTAGATTTCTTTTGATATTTTTATTGCTAACTTCGGATTGTTCATGATAATCTATCTA<br>**TGTAAAC*G*GTTACA**TAAACAAGGAGGAGCTGTTttg…	+6	[25]
resA[b]	CTCAGAGCACTGGCGGATCACGG**TAAAAAC*G*CTTTCT**AAATTTCACATAACCTTCAAAAAGTAAGAAATGTGAAATGAACGTGCAATGATATAATTGAATGGATCTGCAATAG<br>GGGGAAGGGGGCAgtggacaatgaagaaaaaaaggcgtttattcattcggaccggcatccttctcgttttaatctgcgcactcggctataccatttacaacgctgtatttgccggcaaagagagtatatccgaaggtccgacg<br>caccgaattttgtcctagaggatacgaatgggaaacgtatcgagctcagtgacttaaaagggaaaggtgttttttttgaatttctggggtacatggtgtgaaccgtgcaaaaaagagtttccttatatggcaaaccaatataagcatttt<br>aaaagccaaggtgttgaaattgtcgctgtaaatgttggggagtcaaagatagcagtacataattttatgaaatcctacggagtcaatttcccggttgttctggatacagatcgccaagtgcttgatgcctatgacgtatctccgcttccg<br>acaacctttttgatcaatccggaaggaaaagttgtcaaggtggtgaccggcactatgacagaaagcatgatacacgattatatgaatctcataaaacccggagagacttcgggatgaagcaagtcaaatgtgaatgcggacatata<br>aacccggtcggaaccgttttatgcgaatcgtgcggaagagctcttcaggaaactcaacctccgctcgctgatatgagatatgacggaagcgcaagacgttcgcaaacctataacaaaacgatcatcgacaaaatttggaatttctttt<br>cctcggttaaggtgggaatatggcttatcgtcataacccttgccgcatcagcgttcggcaccatttttccccaggaagcctatctgcctccgggcgcacaggctgatacttattacaaggagcagtacggcacgtttggccagctttatt<br>atttgctcgggtttcatcatttatatggttcttggtggtatttgctgctcattgcctcgatcggcatttcactcgtgatttgcagccttgatcgggtcattccgttgtatagagcgttaaaaaaccaaggtgtcagaaggagcccggccttttt<br>aagaaggcagcgcttgttcagtgaaacagttactgtactgaacggggaatcgaaagaaaagatagttactctattaaagaaaaaaacattatagaatcagagaaaaagagggcagcattcttgctgaaaaaggacgcttttcccgct<br>ggggcccgtatgtcaaccacattggattgatcatctttctgattggtgcgatgctgagatttgtgcctggcatgtacgtcgatgagacgctttgggtcagagaaggcgaaaccgcagcaattccgggtacagacggaaaatattatttg<br>aaaaacaatcaattcagcgtagagacctataacagcaaaacggaaaaaaaggtatttgcagatgccattgacagagtcggggacggtagagtggcaaagaactttcaaactgatgccgtactctacaagagagaaggaaaaatt<br>gtttatggcgagaagccgaagctggaaaaagtaacagaagaagacatccgtgtcaatcagccgctgcgttttgattctttttccgtttaccaggtggattacaaagagaatcagcttgaccaaatggtgttccagctgattgataaaa	*cre1*: -72<br>*cre2*: +1709	[26]

	aaacaaaaaaatcctttgggagcctgaagatcaaccttctggatccagattcagtctatgatct**tggtaac*g*gttaca**aggtggaaatcg…		
rocG	CGCAGGCACTTACACGATTAAAGGCGATGTAGTAAACGGTTTGGGAATCGGCAGTTTTTATGCTGAAACGCAGCTGGTGATTGATCCCCGTTAATCGATAGATTTGTAGTCAGA<br>GACTGAGATGTTTTCAGTCTCTTTTTTTGTGGATTCAAAAGCTGGTACGGATCTTGCATGATGATAAGGGTGAATCCAATGAAGAAAGGAATTGGCGGGCTGATTTTTTAAAGC<br>*G*CTTACATTACAGCCGGCCAAAAAAACAATGAGGTGAAAAAGatg…	+43	[27]
sacP	AGCACATAACGGATTTTGCTTGAAGCGAATATGTTTTGTTTTTTCAAAGCATACTGTTCAACCTTTTGTAATGCTTGAGTTTCCATTGTCTTTTCCTGCTTTCTTTCATGATTGAGCA<br>TAGTATAGCACGATATTGTGAAGTATTTCACAATTAATTTTTACGAATTTTTGTCTACATGTTATATCAAACTATTTAACTAAAAAAATAGTTGACGAAAAC*G*CTATCATGATTTA<br>TGATGAAAGCGTATTCTTAATCTGAATAAGCGGGATTGTGACTGGTAAAGCAGGCAAGACCTAAAATTTGCGTAAATGAAAAAGGATCGCTGTGTCCTTTATTCGTTGGCGAA<br>TTTTAGGTCTTTTTTGTTTAAATAAAAGGGGGAGAAATAGatg…	-19	[28]
sigL	TCAGACATTTCTTTGGCAGCTCCTTTTTCATAAATGTATACGGTGTGATCTCAATCATGTACAATGAAGAAAGGGGAGTGAGCAAGatggatatgaaacttcagcaagtacaagtattaaag<br>cctcaactgacacaggagctcaggcaggccatcacgctgcttggctatcattcggcagaactcgccgagtacattgatgagctctcactggaaaaccctcttattgaacgaaaggaaacagacacaccgccactatcttaccataaa<br>acaaacaaaaacaggatgaatgcacaggaagcgggccttcaattaagtaatccgcaaaaaacattgcaggatgcgttaaaacagcagtcactcgatatgaatctgacaaacactgaaaaaaagatttttaattatctgattcattc<br>gcttgattcaaacgggtacttggaagaagatatagaggaggcagcacggcgattatctgtatcggccaaggaagcggaggctgttttggcaaaactccagtcgttagaaccggctggaatcggcgcgagatctttgcaggaatgca<br>tccttcttcaattacagcggctgccgaacagaaatgaacaagcggaaatgctggtttcagctcattttgatgcttttgcgcaaaaaaaat**ggaaaacgctttca**gtagagacgggaattccgctt…	ND	
sucC	ATTTGACGGTAGATGATCAAATTCCTGAAGCTCTTTATGAAACGGTAGCCGAGATTTTTTCATTCATTTACAAATTGGACGAAAGCGTAAAAAACAAAAAATAGCTAAAATATTC<br>CCCATCCTTCTTTGAAATTTATGTTTTTAATAAAGTAAAAGTTTGAATGTTTAGAAGGATTAAAAGATTTTGCATCGAACTGTAGACAATTCTTCCAGTATTATTATAGAATGAAA<br>GC*G*CAGTCTATTTTAGTTTTGTTACATAAGTTAGGAGGATGGGAAatg…	0	this study
treP	AAATTGGGAGAGATCGGTCTGTCCTGATTGTCTTCAAGCTCTTTGATGAGCTGAAGCGCTTTGTCGCGTTTATTCATAGAGGCTCACCCTGCTTTTTAATGTCATATTCAATAAGT<br>GCTTGTGACTTTTAGGGTAACCTTGTCTGTTTCATATATTCCAGACTAGGATGAAAGC*G*CTATAAAAAAGTGTTGACTACCTGTATATACAGGAATACAATATGATTATAAGTTG<br>TATATACAAGTTATAAAAACGGATACGGAGGGGTTGGCatgggggaactgaacaaatcggcacgtcagattgtcgaagcagtcggcggtgctgaaaatattgcagcggcaactcattgtgttacacgtttgcgttt<br>tgctttaatagatgaaagcaaggttgaccaagagatgcttgatcaaattgatgtggtaaagggatcattctcgacaaacggacagtttcaggtcgtaatcggccagggaactgtcaataaagtatatgctgaactggttaaggaaac<br>ggggattggcgagtcaacaaaggatgaagtgaagaaggcctcagaaaaaaatatgaatcctttgcagcgtgctg**tgaaaac*g*cttgca**gatatttttattccaatattgcctgcg…	+372	[29]
uxaC	GCTTCCTTGATGAAAGAAGCAAGTAATGTACGATATTTGACACATATTAAAGGACTTTTCAGCCTGGCTTACTAATCCTTTTAATGAATCTGGCATTGCCAAAAGCATTCCTTTCC<br>GAGTGCTTTCTGGCAATGTCTTTTTTTATGTTTATGACAGCGTTAACATTTCATTTTCAAAAAAGAGATTGATCCCAAAGGAATATAAAGGTAAAATAAAAACAAATCAAAATGT<br>TAACGTTAACATTTTGAAATAGAATGAAAGACGGTGAGGACatggaacccttcatgggcaaaaacttttttattgaaaaatgaaaccgctgtcagcctctatcacaattatgcgaaagacatgccaattattgatta<br>ccactgccacttaagtccgaaagaaatctatgaaaataagacatttcaaaacatcacggaagcctggctgtatggtgaccattataaatggcgcatcatgagagccaatggaattgaggagacatacattaccggagatgcgcctg<br>atgaggagaaatttatggcttgggcgaaaaccgtgccgatggcgattggcaacccgctctacaactggactcacttagaattgcagcgtttttttggcatttacgagattttaaacgagaagtccgggtcggctatatggaaacagac<br>aaataaactgctcaaaggggaaggcttcggggcaagggatttaatcgtgaaatcaaatgtaaaggtcgtgtgcaccacagatgaccctgttgattctcttgaataccatttattgttaaaagaagacaaagactttcctgtcagtgtg<br>ctgcctggatttcggccggataaaggacttgagatcaatcgtgaaggctttcctgagtgggtgcaggcgcttgaagatgccgctgccatatcgattacaacctatgatgagtttttaaaggcgttggaaaaacgagtgcgattcttcca<br>ctcagccggcggcagagtttctgatcacgcgatagatacaatggtatttgccgaaacaacaaaagaagaagctggacggatttttctgacagattacaaggaacagaggtttcttgtgaagatgagaagaaatttaagacatatac<br>gcttcagtttctttgcggtttgtatgcggagctcgattgggcgatgcagtttcatatcaacgctttaagaaacacgaatacaaaaatgatgaaaaggctcggacctgatacagggtatgattcaatgaacgatgaagaaattgctaag<br>cctttatacaagctgctgaactcagttgagatgaagaaccagctgccgaaaacgattttatattcactgaatccaaatgataactatgtcatcgccagcatgatcaacagttttcaggacggtattaccccgggaaaaatacaattcg<br>gcacagcctggtggtttaacgatactaaagacggaatgctcgaccaaa**tgaaagc*g*ttatca**aatgttggc…	+1237	[30]
wprA	AAAAAGAAAAATCAACAGGAGGCCTGTGGGTCGGCTTCGTCATTGTCCTTTTGCTGACAGTGCTGCTCGGTCTGCATTTGCCAATTGGTTTTCAATTGTTTTAATAGAAAAACCT<br>ATGAACCCGGCTCTTTGATAGAGCTGGTTTTTTTTATATTTATCCCCTCATATTCCAAATCATTTAAAATAACCTTAAAATTCCCTGTAAGC*G*GTATCTCGTCCTATGAAATTATGA<br>TACCTTCAAGGAGATTCATTATTTTGCAGGAGGGATAACatg…	+43	this study
xsa	TGAAGTTGTCCCGTTATTAACTGAGGAATTCAAACAGCTGAATATACACTCATAAAGGAAATCCCGGACTTTAAAAGTCCGGGTTTTTTCATATTTATAAATACATACGTACAAA<br>TATAAAAACAATAGAAAATGTCGTTGACATGTACGAACATATATAATATGGTTACTTTAAAAGC*G*CTTACATTCATGCTGACTCCTCAGGCAAACGACAGATCACATATTTGACT<br>GTGGTGCTTTTCATATCCGTATGAAAGGAATGATCAGAGGCatg…	+7	[1]
xylA	CATAGTACATAGCGAATCTTCCCTTTATTAAATCTAATGTGTTCATAAAAAACTAAAAAAAATATTGAAAATACTGACGAGGTTATATAAGATGAAAATAAGTTAGTTTGTTTAA<br>ACAACAAACTAATAGGTGATGTACTTACTATATGAAATAAAATGCATCTGTATTTGAATGAATTTATTTTTAAGGGGGAAATCACatggctcaatctcattccagttcaatcaactattt**tggaa<br>gc*g*caaaca**aagtggtttacgaagggaaagattcgactaatcctttagcatttaaatattataatcctcaagaagtaatc…	+144	[31]

xynP	ACCACTAACCAATTCTTTATTTCGCATAGATCATCTTTGTCATGTGTTTACACAAGAATCTGTTTTGGACTGAGACACTGAATATAACAAAACAGTCAAAAATCAATATTTAATTC<br>ATTCTGTTTAAACTTTCTGAAAAAGATGTTGAAAAAGTCGAAAGGATTTTATAATATTAAGTCAAGTTAGTTTGTTTGATCAACAAACTAATGAAATGAACTTTCATTATCCACA<br>AGATAAGATCTTACTAATATGGATGGAAGGGGGATAAGAGGGTCGATTTAATGGAGCTAAACAATATAAGAAACGAAAGAATGAGGCTTTTATCCTTTTCCAGGTATATAAGG<br>TATGACGAAAAAAGATGAATTTTTTGCTTGCTGATCACCATATTTTTTTGCACTGTTT**TGAAAGCGCTTTTA**TAAAAGTTTACTGACCGTAAATTCGAGAAATTGTTATACCGTTT<br>CACACTGTTCCATATTAAAGGATCGAAAAGGGGAGAAATCatg…	+230	[32]
ycbP	GAAGTGATGACGGAGGGACAGGAGAAGTTTTTAAATTTATTCATCTAAAAACAGCAAACATAATGGGCTGTTGGGCATCAAAGCAGCGTCCGGTGCAGTACAAGCTTCACGTT<br>TCCGCTGTCGAAGCGCCGCAGCCTCAATTCCTGGATTCACATCATTGACCTCTCCCCAGCAGATTTGCACCCGCCTCCGAAAAGAAATAAGGTTTAACTTTTTACATTTGAGGAA<br>TTATACATAACAAATCAAATGACAAAAGGAGGAATTGTAatgacacatgtgaaagcgctcgctattaaaggtattatgacgattattgtgt…	+30	this study
yckB	CCTCTTGACCATTTCTTCCGGTATTGGCATAGATGAGTGACGGGTCTGCTGCATCCTTCACTTTCACATGCGTTTCCTTGGTACGTCTGGCATTCATCCTTGGACAATTCCTTTTTT<br>CATCACCTCTTCCCTTCATAGACGCTTTTAAACACCCACCCATGCTTTTTGTATACACAATTCGAACTTTTTGAAAACGCGATCAACATATTGCAACCCCTTATGTTCAATGCTATC<br>TTTTTAGTTGATTAACAGATTGGGGGAACTATAGgtg…	-48	this study
ycsA	CCGAATGCACTGGCTGAAGGAATTGCCGCAGCACTGCGCTTCGATTTCACCGGTGACCCTGAAGCGGTTGAACTGCAAGCGCTGATCGAAGAAAAAGGATACAGCGGCGTAC<br>TTCAAGAGGTGTGCGGCATTCAGTCCCATGAACCGTTGCACGCCATCATTTTAAAGAAACTTAATCAATAACCGACCACCCGTGACACAATGTCACGGGCTTTTTTACTATCTC<br>GCAATCTAGTATAATAGAAAGCGCTTACGATAACAGGGGAAGGAGAatg…	+67	this study
gmuB	TCATAAACAAATTTTGTAATCAGGATTTTACGGTTCCTGGTAGACACCCTCAAACCATATTATTGAGGTTATACAAGTGATAATAGCTATTTAATTGATTCGTTTCCGTTGATGAG<br>TGTACCACATTATGAATGAATCTTCCATAGAAAAATTTATAAAACGGTGAACCGGAAAAAAATATGATTATATTTATTTTATAAAAGTATAGACATTTAAAATTAAATGACTATA<br>ATAATCAATGTAAGCGTTTTAATAACAGGGGGGATAGCAGttg…	+6	[33]
ydzA	ATTTTTCTGCCTAATTCCCTCATCGACAAACGGCTGTCCTTCTTCAGCTCCTCAATGATATTCAGATCAATCTGGTCAAGTTTCATTTCAACATCCTTCTTTTTTGATTTTGTACACAT<br>TATCTCGGGTATTTTTGTAAATGACAAGTACAGTTCCCTAGAAAAGGCATGTAAAAATGAATGTTTTCCGAACATTTTTTGAAAGCTGTCATATGCCCCCCCGGATTGTTTATAG<br>TATAAAATGAAAACGTGTCCACAAGGAGGGCGATTTatg…	+9	this study
yesL	TGTGGTCAATATTATTTGTGACAGGGATTGTGACGGCTTGTCTGTTTGCTGGCGTATCCGTATTGATGCGGATGAGATTTCCTGACAAAAGTCGGCCGGAATGGATGTTGGCCG<br>GGCTGATCGTCCTTGGTGTGTTTGCGATCTGGTACAGCCTCGTGTATGTACGCGGCTGGGAAGGGGCTGCGCTCGGAATGCTTGGATTCAATGTCATTTTCGGAGCCATTGCC<br>GGATATTTGATCGATAAGGCCATCCGGCGTTACAGGAAAAGATAAGGCTGCTGCCGTCTCTCAACAGGTCGAAATTTTCACAGACCGGCAGGAATTTTCTTTCGAAAGGAGAA<br>ATACATATTACTTTAAAATGAAAGCGTTTTCCAAAGCAGGAAGGTGatg…	+125	this study
yfiG	AAGCTGCCGAACTGCTGAAATCAACGAATTTGTCGATTAAAGAAATAGCGGAAGAAATCGGTTTCTCAGTACATTATTTCACTCGCGTCTTTTCCGCTAAAATCGGCAGCTCTCC<br>AGGGCTTTTCCGCTCCCTTTATAAAGACTCAAAAATGACCGCATTTCAAATCAATAAACCTTTTCAAAAAATCGAACAGGCCAAGCCGCACGAATAAGATGTCCTGAAATGACA<br>AAACAATGTCTTCATATGATAAAGACGCGAGAAATCTCGTTGTCTTATAATCTTTTTAGAAAGCGGTTACAACAAGGGGTATTGCTGAAGGAGGGATGCATTGGCTTATGCTTG<br>GTGTCGGGTGCTCTGCCATTCTTGAAAGAAGGGGAAGAAAGCCgtg…	+38	this study
yisS	AAACAAAAAATAGTGCTCCCGCCTGAGCTTGTAGTCAGACATTCTACATCACCGCTCAACACATGAGCCCGCTAATGAGCGGGTTTTTTCATTATGACAGTTGAAGCCAAAAAA<br>ACATATTGCGTGTAAACCAAAGGCGGTGTAAAATATAACCATCTTTTGAGAAAGCGCTTGCGCAAAATGGCAGAGCGGATAGAACCGCTGTTTTTTTAGCTGTTAAGAGAAAA<br>CGCTTTCTCAAAGCGAGCCCGAAATGAGATAAAGGAGAGGATGGGACgtg…	+74	this study
yjmD	CTCATATTGCCAAAATGTACAACGGCCTTGATCAAAAAAGAAAGCTGGGGCATTACGTTTGCGCGATCAATCCATCCTTTTTTACTGACTGGGATACGTTTTTAGAGCAGATGGA<br>TGCCATGATTGATGAACTGCAGCAATCACCGCCGGCTGTTGGATTCGAAAGAGTGTATGTGCCCGGCGAGATCGAGCAGCTGCATGAAGAAAGAAATAAGAAAAACGGAATT<br>TCTATCGCCCGGAGCGTGTATGAATTCTTAAAAAGCAGGTGAGTAAAatgaaagcggttcaagtgcgaaaagcgtatgatctggtgacagcgga…	ND	
ykoM	ATTATTCTGAAAAACTCTATCAGATAGGGGTTTATAATAAAGAGGTCATGAACAAAATGACGATTTCTGAGCTTCGGAAAGAATACGCTTATTTCTTTCGAAGCATTACAAATCA<br>TAAAAATTATCCTTATACAAGATAATGTATAAGGATTTTTTTGCTTTTAGAAGGAATTTCTCGAATTATAGCGAATACGTATAGTAAGGGACTGAACTCTTATTATATGTAAGGG<br>TTTTCATTATTTTTTTATAAAAATCCTATATACGAGGTAGATatgatgagattatcgtttaacgaagaagaagttgagcgtgcgatgaatttgtacagagttttttgcaaggggctttcaaaaagtgtgtccg…	+150	this study
yncC	TTTCGCGATTAAACATCATCATTATAACGATATGGACATTTTTTGTGAATGGAAAACTTCAAGGGAACTTATAACACTCCAAATTGCCAATAGTTAACTTCACAACGAGATGATC	+84	this study

	AAAACATCACCATCACATAGCGGAAATAAATAATCCTGAAAATAATATTGACTGAAAATGCTTTTAGAATTATGATAATATGAGAAATGACAATAATGTGACAAAGACATTACTACAACATAACAACTAATAGCTTAGGTTTATTCTATTGGTTAAAAATGTAAAC*G*GTTACAAATCCAGAGGCGAAAATCTAAGATAGAGATGACTTTAGTTTTGACTCAATATCCACACTGAAAGAGCTTATTAAGTATTCTTAAAGGCAGGGGAAatg…		
yojA	CAAAACGCAATAATGGCATGTGTACTCCTCCTCTTATGAAGTTGTTATCCGTCTGTTCTCATATTGTCTCATTTGCTTCCAGACAATGGAGAGATATAATGATTGGCAGTCAACTTTAAAAAACATAACATATAACCAAAAGTTAATCAACATATAACTAATAAATCATTGGGTTAGGCGGTAGGTAATCGAGACAAGCTCATAAAAACGATTGACAGTCATTGATTAGGAGATTAGTATGAACATATTAGTTGATCAAAAGATAACAATCATATAACCAAGACGTTTTTAGTTATTAATTGAAAGC*G*CTTTCTGTTCATCTAGGCATGAAAAGCGCCAAAACATTGTCGCGAGAATCATATAAAACACTTTTGGAGGGGTACCatg…	+57	this study
yqgW	TGATTGATTTTATGTCCCTCGCCGCCCGGATCAAAAATCAGACATTGATCATCACTGATGAGAAAATATGCGTTCGCTTGAATCGGCCCTACTGGCATTCGTCTCCATTTCACTATTATCACCTCTTTCTTTTTTATGCTCTTATTATGAGGGTTTTTGAAAAAAGATTCAAACAATCGCAATGAAAAC*G*CTATCGACAAACCTATAAAAAACAGGTAGAATAAAACTGGAAAGAATTGCAGGTTTTTCAATGTAAAGGGGGCTGTGTTatg…	-39	this study
yqgY	TTGGATATGAGTAAAAAGTTAATTGTTGTTCCATTCGTTTTTTCCTCCTCAGTTAACCATCTCTTTGTAGAATAACTACCCGGATCATTTTTCATTTAAACCATTTACAGAACTTTTTTCGGATTATTTTTTAAAAAACATTCATACAATAGAAAACACAATCAAAGGGACAAATTCCATTTGTATATTAATAATGAAAAT*G*TTTACATTTCTAGGTGTGATCTATTATAATAACAGTCAGAGGGACTCTCGGGAGGGGATAAAAatg…	-38	this study
yrpD	CATTGAATGTAAATGAAAAAGACAATCTCCTGAAGAGAAACATGAACTAAGAAGCGGGCTTAAAAAGCCCACTTTTTTTCTAACAAACAATTGAGTTATACACACATATCTGATTACAGTGTTTTCTCTCTTTCCCCTCTTATTTCTCCCCCCTCTCTATAGCTTTTCTATTTCAATAAAACACAAGTCTCGATTGAAAAGTTATCGTAACATTTACACTTCACAGGAGATTTTTCACGATTGCGAATCGACCGTTTGCCAGATGCACAAAGTCATAATGTGTAAATTCTGTATCTATGTGCATGTTTATTATGGAATTATTTTCATAATGTTTTATAATACAAGATTGATAGC*G*TTTTCTAATTATAATGATAGGGGGAAATAATGatg…	+127	this study
ysbA	CTTTGTCGTGAATACTGAATATATTAAAGAAATTCAGCCATGGTTTAATTCAACGTATAATCTGATCATGAAAGACGGATCGAAAATACCGGTCAGCCGGACATATGCGAAGGAATTGAAAAAGCTGCTCCATATTTGATGGGCGGCTTTTTGCATTTCAGCCATTTGATTCTGCAACTCAGACCCGCTATACGGCATGTTATCTTGTAAGC*G*CTTTATAACAGGGAATTCTTATTTATAATGAGGGTACCAAAGAGAAAGAGGTGAAGAAAatg…	ND	
ysfC	GCTTGATATGTGTCTGACGGAGATCAGCCCGGGAACCCGAAACGAATTTCCGCAAAGAGTATTAGGAAAGGCGCTAGAGCATCAGGAACTTATGAATACGATACGGACTTTCTTTCATCACGATTTGTCTTTGAAACAAACCGCTGAAGACATGCATATACACATCAATACATTGCGTTATCGTCTGGCTAAGGCTGAACAGCTGACGGGATTACGTTTTGACCGTACCGAGGACGTCGTCACGATGTACGTCGCACTCTATTTTTTAGATCAAGATACAAAATAGAGAGAGTTTCAGTTTCATTTTTTGTATGTTTCTCCATGGAAGGCTTTGCTTTTTTTCTTTTACAATAGATTGAAAGC*G*TTTTTTGACAGGGGGATGGGTGAAatg…	+196	this study
ytkA	AATGAAGGAAGCGGTAGAGATTACAGAAATACCTGCTGCGAATGAAAAGCAGTGCGGCCAAGCGAAGCTTCATGATCTGGAAGGCGCTAAACGTTTAATGCGTTTCTGGCTTTCACAGGATAAAGAAGAATTGCTAAAAGTATTTGGCTAAAATAGAAAGGACCTTTTTGCGCTTAAGCAAAAAGGTCTTTTTTGTGACGTCTCATATTTTGTGTACCATTTGCTGTAAGC*G*TTTGCTACTATTTAGGTAAACGATGAAAGGGTGATGGCatg…	ND	
yugN	CTTGCCCTTTTTACCGATGCGGTACATCGGATTTTGCATTTAAAACGAACCCATACTCTTTTCACTAAATTAGGACTCTATGCAACGGTTGTTTATGTGTCTTGGGATTCAGCTGTATGGCTTGCAGCGGCAATGGCTGTTTACTTCTTAATTGAATGC*G*CTTTCTTTCCTGTTGGCCGCGCGAAAGAAACAACGATTTCCATGTAGGCGCCTCTGAATGAAGTCTTGTTCGGCGGGACAGGATAAGTGAAAAAAGGGGCATTCTGatg…	ND	
yulD	CCCGGAATGATTTCTTAAATCAATTAACGGCAGACATGAGTGGAAAAGCGGTATATGCAGGGCCGATAGAGGCAACTGCCACCGGAAATCTTTTGATGCAAATGATTGCTGCCAAAGAAGTCAAAGACATAAAAGAAGCCCGGCAAGTCGTCAGAAATTCCTTTCCGATCAAAGTGTTTACACCTAAAGACATTGATAGAAGCACGATCATTCAGTCATTTCAGCAAACTGTTTTGAAAGC*G*CTATCTAAATGATGAAGAGGTGAAAAGGTttg…	ND	
yuxG	GGCATTCAGGATATCGCAGCTTCGGCGGAAGAACAGCTTGCGTCCATGGAAGAAATCAGCTCTTCAGCGGAAACGTTGGCAAACATGGCGGAAGAACTTCAGGACATTACGAAGAAATTTAAAATAGAATCATAATGAAACGAGAAAGCGGCATATCTGCTGCTTTCTTTTTTTGTTAAAAAGCAAAAATAAACAAAAACGATTCAAAAACAAACAGAAATCATATATAATGAAAAC*G*GATACAAATAAAAGGGTATGGAGGAATATCAAATatg…	0	this study
yvdG	ACTGATTTATTTCTTTAATCAAGGGAATGTGGTTCAACACGTCAGTTTACGAGAGTTGAATATTGATAGGAATAAGAAAATCTGTGATGCATGGACAGAGCAGCCGCTACAGCATCACGATGTCATTGCCGTTCAGCCGGGAGAATTTTTGATTTTGAGTGCTGCCGCCCCTGTATAACATTCTGTTATCGGTAACAAATCGAAAGTTGTTGTAACC*G*CTTTCTATAGGACAATACAATTGTTATAGTTTCGTAAATAGAAGGAGGAAAAGAGatg…	-28	this study

yvfK	TTCATATTGATATACATGAATTATGTAAAAACGCTGTTCAATTACTGCTTGAACAAGTGCAGGACAAGAGAAGAACGGTAAAAACATTATATGTGGGCGCAGAATTAATCGTCA<br>GGAAGAGTATGAATTAAGGATGACTTAGGACACTAAGTCATTTTTTATTTAGGTAAAAAAATTTACTCTATGAAGTAAATAGTTTGTTTACACATTTTCTCAGGCATGCTATATT<br>ATCTTTAAAGCGCTTTCATTCCTACCGAAAGGGTGACAATCAatg…	+5	this study
yxlH	CACGTCAACTACGAGCCATCCATTATGGGCGGGCTGAAGGAAGCGAAGCAAGACGGAAAAGACCATACGCCGCATGTCGAAGGTGATGTAAAGCGTGAAGCGATTGACAGA<br>ACAAACAACTTCGGACAGGCTGGAGAAACGTACCGGAGATTTACTGAATTTGAGCGGAACGAATTGATTACGAATTTGGTGAATACGCTTTCTACGTGCCGAAAAGAAATTCA<br>GGATCAAATGATTGAGAATTTCACAAAAGCTGATCCTGACTACGGAAAACGCGTAGCAGAAGGGCTAAAAAAGGTCTCCGAAAACAACAGCAACGGGCCGATCGGCACAACT<br>GAAACGGAACAGGCTGCAAAGCAGGCTGAACAGGAGAGTCATCCGTCTGATCCGTATTAACAACAGCGAACAGGGCTTTTTAGAAGCCCTGTTTTTTATTTTTCTATTGAAA<br>CGCTTTCAATAATTGTGTTGTACTTGGAAGGAAAGGGGTGGAGAGATACGatg…	+260	this study
yydK	GCACCAATCGAAGTCTGGTAGCGCAGCGAGAAGCCCCAATCACATTCTCTTCCCCGCCTACGGCCTCTAAAATGTCTTTTGCTAGTCTCGTATAATCTCTCACTTTCCCCGACATT<br>TCACCCACCCCTTCGTTTTGGTTACGCTTTCATTATAATTGTAACGGTATAATTTATCAATTCCGAAACAAAATATTTACGCACTAACTATCATTGTAAGCGGTTTATGCTATAATT<br>TTGACAAGTGAAACTAAACGAATAAATGAGGAATGCGTatg…	-21	this study
yyzE	ATGTTTCAATTTCCGTTGCGATTTGCTGGTATTTTAACATACGCATTCCTCATTTATTCGTTTAGTTTCACTTGTCAAAATTATAGCATAAACCGCTTACAATGATAGTTAGTGCGT<br>AAATATTTTGTTTCGGAATTGATAAATTATACCGTTACAATTATAATGAAAGCGTAACCAAAACGAAGGGGTGGGTGAAATGTCGGGGAAAGTGAGAGATTATACGAGACTA<br>GCAAAAGACATTTTAGAGGCCGTAGGCGGGGAAGAGAATGTGATTGGGGCTTCTCGCTGCGCTACCAGACTTCGATTGGTGCTGAAGCGGTCTACCCCTAAAGCGAAGGATA<br>TCGTGAAATCGATGCTACCATCCCGGCGTCATTACGGTTGTCatg…	0	this study

[a] *cre* to TSS distance calculated from conserved (mostly) G residue (in italics) in the middle of the *cre* box sequence to TSS. ND, not determined due to lacking TSS.

[b] *kdgR cre* and *resA cre2* boxes were not included in the sequence and position analysis since the microarray chip probes were synthesized upstream from them, thus expression fold changes for these genes were not simply calculated as ratios of the amounts of transcripts of the regions downstream of these *cre* boxes.

References

1. Raposo MP, Inácio JM, Mota LJ, de Sá-Nogueira I: **Transcriptional regulation of genes encoding arabinan-degrading enzymes in *Bacillus subtilis***. *J. Bacteriol.* 2004, **186**:1287–1296.

2. Ould Ali N, Bignon J, Rapoport G, Debarbouille M: **Regulation of the acetoin catabolic pathway is controlled by sigma L in *Bacillus subtilis***. *J Bacteriol* 2001, **183**:2497–2504.

3. Grundy FJ, Turinsky AJ, Henkin TM: **Catabolite regulation of *Bacillus subtilis* acetate and acetoin utilization genes by CcpA**. *J. Bacteriol.* 1994, **176**:4527–4533.

4. Nicholson WL, Park YK, Henkin TM, Won M, Weickert MJ, Gaskell JA, Chambliss GH: **Catabolite repression-resistant mutations of the *Bacillus subtilis* alpha-amylase promoter affect transcription levels and are in an operator-like sequence**. *J. Mol. Biol.* 1987, **198**:609–618.

5. Le Coq D, Lindner C, Krüger S, Steinmetz M, Stülke J: **New beta-glucoside (*bgl*) genes in *Bacillus subtilis*: the *bglP* gene product has both transport and regulatory functions similar to those of BglF, its *Escherichia coli* homolog**. *J. Bacteriol.* 1995, **177**:1527–1535.

6. Monedero V, Boël G, Deutscher J: **Catabolite regulation of the cytochrome c550-encoding *Bacillus subtilis cccA* gene**. *J. Mol. Microbiol. Biotechnol.* 2001, **3**:433–438.

7. Yamamoto H, Murata M, Sekiguchi J: **The CitST two-component system regulates the expression of the Mg-citrate transporter in *Bacillus subtilis***. *Mol. Microbiol.* 2000, **37**:898–912.

8. Jin S, Sonenshein AL: **Transcriptional regulation of *Bacillus subtilis* citrate synthase genes.** *J Bacteriol* 1994, **176**:4680–4690.

9. Gomez M, Cutting SM: **Identification of a new sigma B-controlled gene, *csbX*, in *Bacillus subtilis***. *Gene* 1997, **188**:29–33.

10. Winstedt L, Yoshida K-I, Fujita Y, von Wachenfeldt C: **Cytochrome *bd* biosynthesis in *Bacillus subtilis*: characterization of the *cydABCD* operon**. *J Bacteriol* 1998, **180**:6571–6580.

11. Asai K, Baik SH, Kasahara Y, Moriya S, Ogasawara N: **Regulation of the transport system for C4-dicarboxylic acids in *Bacillus subtilis***. *Microbiology* 2000, **146 (Pt 2)**:263–271.

12. Schuch R, Garibian A, Saxild HH, Piggot PJ, Nygaard P: **Nucleosides as a carbon source in *Bacillus subtilis*: characterization of the *drm-pupG* operon**. *Microbiology* 1999, **145 (Pt 10)**:2957–2966.

13. Holmberg C, Beijer L, Rutberg B, Rutberg L: **Glycerol catabolism in *Bacillus subtilis*: nucleotide sequence of the genes encoding glycerol kinase (*glpK*) and glycerol-3-phosphate dehydrogenase (*glpD*)**. *J. Gen. Microbiol.* 1990, **136**:2367–2375.

14. Fujita Y, Fujita T: **Identification and nucleotide sequence of the promoter region of the *Bacillus subtilis* gluconate operon**. *Nucleic Acids Res.* 1986, **14**:1237–1252.

15. Wray LV, Fisher SH: **Analysis of *Bacillus subtilis hut* operon expression indicates that histidine-dependent induction is mediated primarily by transcriptional antitermination and that amino acid repression is mediated by two mechanisms: regulation of transcription initiation and inhibition of histidine transport.** *J Bacteriol* 1994, **176**:5466–5473.

16. Grandoni JA, Zahler SA, Calvo JM: **Transcriptional regulation of the *ilv-leu* operon of *Bacillus subtilis*.** *J Bacteriol* 1992, **174**:3212–3219.

17. Pujic P, Dervyn R, Sorokin A, Ehrlich SD: **The *kdgRKAT* operon of *Bacillus subtilis*: detection of the transcript and regulation by the *kdgR* and *ccpA* genes**. *Microbiology* 1998, **144 (Pt 11)**:3111–3118.

18. Tojo S, Satomura T, Matsuoka H, Hirooka K, Fujita Y: **Catabolite repression of the *Bacillus subtilis* FadR regulon, which is involved in fatty acid catabolism**. *J Bacteriol* 2011, **193**:2388–2395.

19. Martin I, Debarbouille M, Klier A, Rapoport G: **Induction and metabolite regulation of levanase synthesis *in Bacillus subtilis***. *J. Bacteriol.* 1989, **171**:1885–1892.

20. Yamamoto H, Serizawa M, Thompson J, Sekiguchi J: **Regulation of the *glv* operon in *Bacillus subtilis*: YfiA (GlvR) is a positive regulator of the operon that is repressed through CcpA and *cre***. *J. Bacteriol.* 2001, **183**:5110–5121.

21. Yoshida KI, Aoyama D, Ishio I, Shibayama T, Fujita Y: **Organization and transcription of the myo-inositol operon, *iol*, of *Bacillus subtilis***. *J. Bacteriol.* 1997, **179**:4591–4598.

22. Resnekov O, Melin L, Carlsson P, Mannerlöv M, von Gabain A, Hederstedt L: **Organization and regulation of the *Bacillus subtilis odhAB* operon, which encodes two of the subenzymes of the 2-oxoglutarate dehydrogenase complex**. *Mol. Gen. Genet.* 1992, **234**:285–296.

23. von Blohn C, Kempf B, Kappes RM, Bremer E: **Osmostress response in *Bacillus subtilis*: characterization of a proline uptake system (OpuE) regulated by high osmolarity and the alternative transcription factor sigma B**. *Mol. Microbiol.* 1997, **25**:175–187.

24. Presecan-Siedel E, Galinier A, Longin R, Deutscher J, Danchin A, Glaser P, Martin-Verstraete I: **Catabolite regulation of the *pta* gene as part of carbon flow pathways in *Bacillus subtilis***. *J Bacteriol* 1999, **181**:6889–6897.

25. O'Reilly M, Woodson K, Dowds BC, Devine KM: **The citrulline biosynthetic operon, *argC-F*, and a ribose transport operon, *rbs*, from *Bacillus subtilis* are negatively regulated by Spo0A**. *Mol. Microbiol.* 1994, **11**:87–98.

26. Sun G, Sharkova E, Chesnut R, Birkey S, Duggan MF, Sorokin A, Pujic P, Ehrlich SD, Hulett FM: **Regulators of aerobic and anaerobic respiration in *Bacillus subtilis***. *J Bacteriol* 1996, **178**:1374–1385.

27. Belitsky BR, Sonenshein AL: **An enhancer element located downstream of the major glutamate dehydrogenase gene of *Bacillus subtilis***. *Proc. Natl. Acad. Sci. U.S.A.* 1999, **96**:10290–10295.

28. Arnaud M, Débarbouillé M, Rapoport G, Saier MH Jr, Reizer J: ***In vitro* reconstitution of transcriptional antitermination by the SacT and SacY proteins of *Bacillus subtilis***. *J. Biol. Chem.* 1996, **271**:18966–18972.

29. Schöck F, Dahl MK: **Expression of the *tre* operon of *Bacillus subtilis* 168 is regulated by the repressor TreR.** *J Bacteriol* 1996, **178**:4576–4581.

30. Mekjian KR, Bryan EM, Beall BW, Moran CP Jr: **Regulation of hexuronate utilization in *Bacillus subtilis***. *J. Bacteriol.* 1999, **181**:426–433.

31. Gärtner D, Geissendörfer M, Hillen W: **Expression of the *Bacillus subtilis xyl* operon is repressed at the level of transcription and is induced by xylose.** *J Bacteriol* 1988, **170**:3102–3109.

32. Galinier A, Deutscher J, Martin-Verstraete I: **Phosphorylation of either crh or HPr mediates binding of CcpA to the *Bacillus subtilis xyn cre* and catabolite repression of the *xyn* operon**. *J. Mol. Biol.* 1999, **286**:307–314.

33. Sadaie Y, Nakadate H, Fukui R, Yee LM, Asai K: **Glucomannan utilization operon of *Bacillus subtilis***. *FEMS Microbiol. Lett.* 2008, **279**:103–109.
